# Effective Coverage and Systems Effectiveness for Malaria Case Management in Sub-Saharan African Countries

**DOI:** 10.1371/journal.pone.0127818

**Published:** 2015-05-22

**Authors:** Katya Galactionova, Fabrizio Tediosi, Don de Savigny, Thomas Smith, Marcel Tanner

**Affiliations:** 1 Department of Epidemiology and Public Health, Swiss Tropical and Public Health Institute, Basel, Switzerland; 2 University of Basel, Basel, Switzerland; Université Pierre et Marie Curie, FRANCE

## Abstract

Scale-up of malaria preventive and control interventions over the last decade resulted in substantial declines in mortality and morbidity from the disease in sub-Saharan Africa and many other parts of the world. Sustaining these gains will depend on the health system performance. Treatment provides individual benefits by curing infection and preventing progression to severe disease as well as community-level benefits by reducing the infectious reservoir and averting emergence and spread of drug resistance. However many patients with malaria do not access care, providers do not comply with treatment guidelines, and hence, patients do not necessarily receive the correct regimen. Even when the correct regimen is administered some patients will not adhere and others will be treated with counterfeit or substandard medication leading to treatment failures and spread of drug resistance. We apply systems effectiveness concepts that explicitly consider implications of health system factors such as treatment seeking, provider compliance, adherence, and quality of medication to estimate treatment outcomes for malaria case management. We compile data for these indicators to derive estimates of effective coverage for 43 high-burden Sub-Saharan African countries. Parameters are populated from the Demographic and Health Surveys and other published sources. We assess the relative importance of these factors on the level of effective coverage and consider variation in these health systems indicators across countries. Our findings suggest that effective coverage for malaria case management ranges from 8% to 72% in the region. Different factors account for health system inefficiencies in different countries. Significant losses in effectiveness of treatment are estimated in all countries. The patterns of inter-country variation suggest that these are system failures that are amenable to change. Identifying the reasons for the poor health system performance and intervening to tackle them become key priority areas for malaria control and elimination policies in the region.

## Introduction

Scale-up of malaria preventive and control interventions over the last decade resulted in substantial declines in mortality and morbidity of the disease in sub-Saharan Africa and many other parts of the world [1–4]. As many as 274 million cases and 1.1 million deaths were prevented by the focused effort of the national malaria control programs and global community over the last decade [4]. Sustaining these gains will depend not only on the availability of resources but also on the health systems performance [5]. Treatment provides individual benefits by curing infection and preventing progression to severe disease as well as community-level benefits by reducing the infectious reservoir and averting the emergence and spread of drug resistance [4]. Understanding the extent of the health benefit achieved with malaria case management has thus critical implications for malaria control and elimination programs.

Current global guidelines for malaria case management require prompt parasitological confirmation by microscopy or RDTs in patients suspected of malaria; pending diagnostic confirmation the recommended treatment for uncomplicated *P*. *falciparum* malaria includes at least 3 days of therapy with artemisinin-based combination drugs (ACT) [6]. Artemether lumefantrine (ALU) is the most commonly recommended first-line treatment across the SSA [4]. The drug provides fast relief of symptoms and enables a nearly 100% clearance of parasitaemia if treated within 24 hours of onset of fever. Despite availability of efficacious therapy many patients with malaria do not have access to it or delay appropriate treatment seeking, and providers do not always comply with treatment guidelines, so patients do not necessarily receive the correct regimen or instructions. Even when the correct regimen is administered some patients will not adhere and yet others will be treated with counterfeit or otherwise substandard medication leading both to treatment failures and potentially also to the spread of drug resistance.

The effectiveness of malaria case management depends therefore on a number of health systems factors that should be taken into account when assessing the impact of control strategies. One important measure of the effectiveness of malaria service delivery is the systems effectiveness [7,8], which measures the proportion of clinical events that are effectively treated by the formal health services that offer interventions by health professionals (including hospitals, health centers, and community health workers). A second measure, the effective coverage [9,10] includes in addition the effects of self-treatment, or of treatment obtained from untrained drug sellers or other informal providers. Although informal providers should comply with national guidelines for case management, in practice compliance in the informal sector is generally lower than with formal provision. Effective coverage is nevertheless higher than systems effectiveness because informal provision adds more providers and therefore higher access into the calculus of effectiveness, even though such provision may be of lower quality.

Supply side determinants of these metrics include access to treatment, diagnosis, staff training, and availability of antimalarials at the facility level [11–16]; on the demand side patient awareness and perception of illness, affordability of treatment and adherence to the drug regimen are commonly considered [17–19]. Analyses generally focus on the limits to systems effectiveness of delivering ACTs to febrile or malaria patients through the public sector with estimates generated for the region as a whole [11,20] and for a given country [12,13,18,19,21,22]. These evaluations reveal large inefficiencies in malaria service delivery across the endemic countries. Illustrative of the magnitude are findings from [23] that demonstrated decay from 98%—theoretical efficacy of ACTs—to just about 38%—effectiveness- by the time the medication is administered. Similarly, low levels of effective coverage were estimated for Tanzania of about 5% [12], Ghana—31 to 42% [19], and Zambia—25% [18].

The geographic coverage of these evaluations remains limited with meaningful cross country comparisons hindered by differences in methodological approaches and adopted framework; in particular the choice of parameters considered for the health system factors varies greatly across the studies. Focusing on public sector and ACT therapy these analyses represent only a fraction of malaria case management services delivered in SSA countries. Frequent stock-outs of ACTs[24], presence of non-ACT antimalarials[16,25], heavy reliance on informal care providers[4] as well as concerns about the quality of antimalarial drugs[26,27] in the region call for a more comprehensive measure of effective coverage for the sector.

In this article we estimate performance of malaria case management in Sub-Saharan African countries focusing on clearance of the infection among malaria patients. In other words, we are primarily interested in the proportion of malaria fevers that are cured by the prevailing health system and further in identifying the sources of inefficiency in service delivery and their relative importance for malaria patient outcomes. We produce a consistent measure of effective treatment that captures both public and private health care providers and treatments with drugs other than the first-line therapy for each of the 43 high malaria burden countries.

## Methods

### Estimation framework

**Fig 1:**
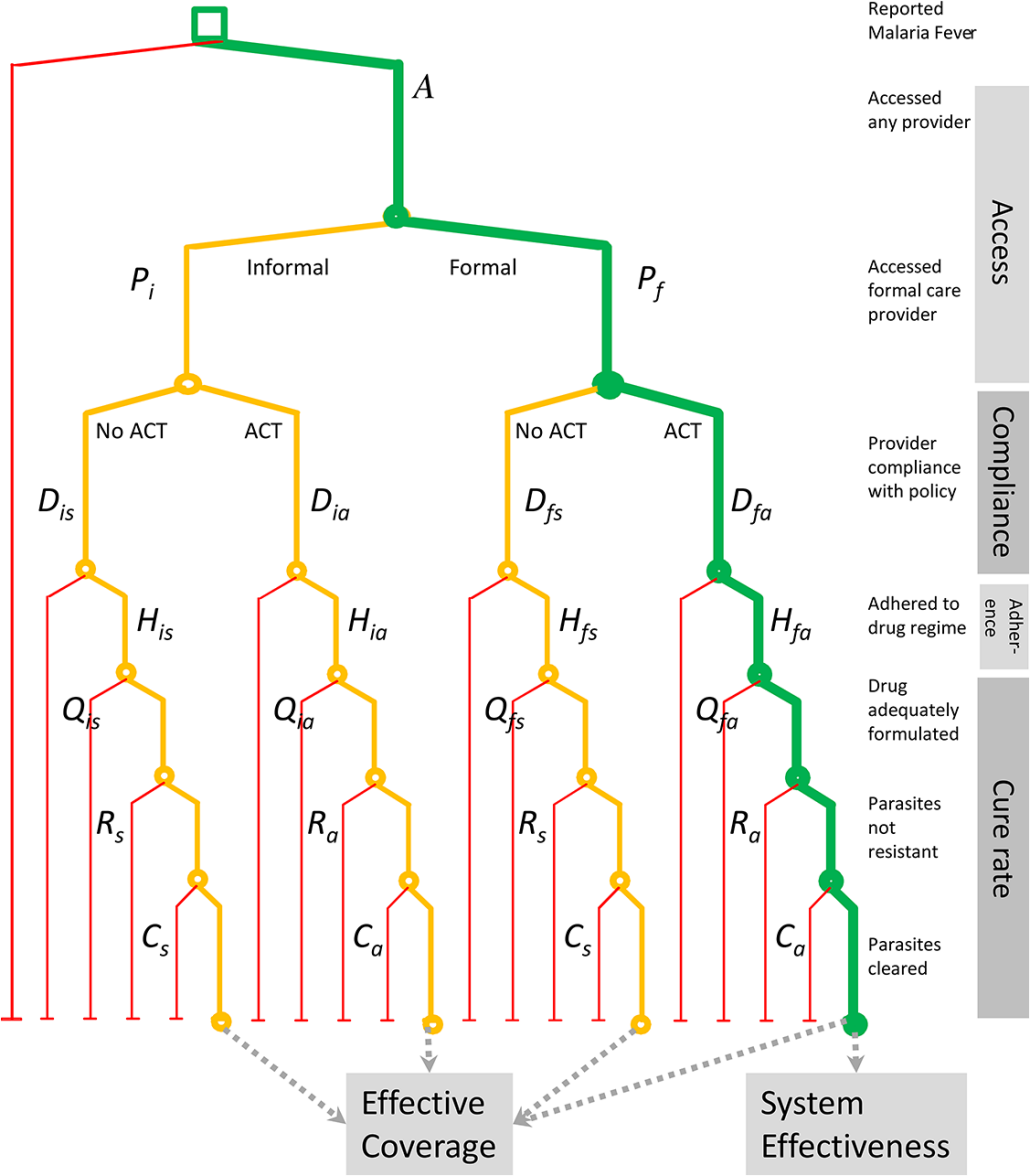
Decision Tree Model for Case Management of Uncomplicated Malaria.

The methodology to estimate the performance of case management of malaria fever is based on a set of indicators that capture the key factors that determine the effectiveness of treatment. The analysis is based on a decision tree (Fig 1) which is an extension of our previously published model for case management of uncomplicated malaria [28]. The entry point to the model is a malaria fever episode, from where the branches move to describe treatment seeking, and then by level of care compliance with the recommended first-line antimalarial therapy. We include adherence with the drug regimen and its cure rate, which in turn is modelled as a function of drug quality, resistance, and clinical efficacy. Combining these parameters, we obtain a measure of effective coverage (*E*) defined here as the expected probability of clinical and parasitological cure for an episode of malaria fever. Algebraically the effective coverage (*E*) is expressed as a function of health seeking (*A*) scaled down with the weighted average cure rate of an antimalarial treatment (*T*_*pd*_) across different drugs (*d*) and providers (*p*) (Eq (1)):

$$E=A(\sum_{p}P_{p}\left[\sum_{d}D_{pd}H_{pd}T_{pd}\right]),$$ (1)

where $\sum_{p}P_{p}=1$, $\sum_{d}D_{pd}=1$

*A* is the proportion of malaria fevers seeking care;

*P*_*p*_ is the proportion of care-seeking malaria fever episodes accessing provider *p;*

*D*_*pd*_ is the proportion of malaria fever episodes treated by provider *p* with antimalarial therapy *d*;

*H*_*pd*_ is the proportion of fever cases treated with drug *d* by provider *p* that adhere with the regimen;

*T*_*pd*_ is cure rate of antimalarial therapy *d* obtained from provider *p;*

Where the cure rate (*T*_*pd*_) accounts for treatment failure due to suboptimal quality of medications (*Q*_*pd*_), resistance (*R*_*pd*_), and clinical efficacy of the antimalarial therapy (*C*_*d*_) (Eq (2)):

$$T_{pd}=(1-Q_{pd})(1-R_{d})(1-C_{d}),$$ (2)

where *Q*_*pd*_, *R*_*d*_ and *C*_*d*_ are ≥ 0 and ≤ 1

*Q*_*pd*_ is the proportion of antimalarial treatments *d* obtained from provider *p* that are counterfeit or of substandard quality;

*R*_*d*_ is the proportion of episodes caused by parasites resistant to drug *d;*

*C*_*d*_ is the parasitological failure rate of sensitive parasites for drug *d;*

As an extension this framework can be applied to evaluate the probability that the health system treats a fever effectively and in line with the official policy (the right hand branch in Fig 1). Here the systems effectiveness refers to the performance of the formal health system, and is therefore:

$$S=AP_{f}D_{fa}H_{fa}T_{fa},$$ (3)

where *f* corresponds to formal health services and *a* to the recommended first-line antimalarial treatment (see Table 1 and Fig 1).

**Table 1:** Summary of Definitions, Sources of Data and Key Assumptions on Malaria Service Performance Indicators.

Model Input	Nota-tion	Definition	Data Source	Core Assumptions	Additional comments
**Access to any care provider**	*A*	Proportion of <5 fevers for which care is sought	DHS, MIS, MICS	Patterns in health seeking for febrile illness are representative of malaria	
**Access to a formal care provider**	*P*_*p*_ *|p = f*	Proportion of access for which care is sought from formal care provider	DHS	Patterns in health seeking by provider type for febrile illness are representative of malaria	Formal provider—treatment based on diagnosis by a medical professional- including hospitals, health centers, community health worker, etc.
**Access to an informal care provider**	*P*_*p*_ *|p = i*	Proportion of access for which care is sought from informal care provider	DHS	Patterns in health seeking by provider type for febrile illness are representative of malaria	Informal provider- treatment based on self-diagnosis- including pharmacies, shops, street vendors etc.
**Compliance**	*D*_*pd*_ *|d = a*	Proportion of treated <5 malaria fevers that receive first-line antimalarial therapy (ACT)	DHS, MICS, WMR, [36,59,74–79]	All malaria related fevers for which treatment is sought are treated with an antimalarial	First-line antimalarial therapy defined according to country policy
**Non-compliance**	*D*_*pd*_ *|d = s*	Proportion of treated <5 *malaria* fevers that receive antimalarial therapy other than first-line medication	DHS, MICS, WMR, [36,59,74–79]	All *malaria* related fevers for which treatment is sought are treated with an antimalarial	Non-compliant treatment with therapy other than the first-line medication is proxied with SP
**Adherence**	*H*_*d*_	Proportion of treated <5 malaria fevers that adhere with the drug regimen (*d = a*: first line ACT regimen; *d = s*: other)	DHS, [49–56,80–82]	100% failure rate for partially adherent treatments	Adherence with the drug regimen is defined in terms of days of treatment
**Cure rate**	*T*_*pd*_	Efficacy of antimalarial drugs taking into account clinical and parasitological failure rate *C*_*d*_, resistance *R*_*d*_, and incidence of counterfeit and sub-optimal formulations *Q*_*pd*_	WMR, [41–45,83–85], [46]	100% failure rate for treatments with sub-optimal or counterfeit antimalarial drugs	Clinical efficacy, levels of resistance and prevalence of sub-optimal or counterfeit antimalarial drugs are assumed to be the same across SSA countries
**Effective coverage**	*E*	Proportion of <5 malaria fevers cured	Derived	Service performance indicators relate linearly to treatment outcomes	
**System effective-ness**	*S*	Proportion of <5 malaria fevers cured by the formal health system.	Derived	Service performance indicators relate linearly to treatment outcomes	

### Data

The definitions, data sources, and key assumptions for each parameter defined in the model are outlined in Table 1 and further by country in S2 Table. Service indicators related to access and type of care provider, compliance and adherence are measured primarily from the Demographic and Health Surveys (DHS) [29–31] and the Multiple Indicator Cluster Surveys (MICS) [32]. These nationally representative household surveys are used extensively for research in low- and middle-income settings [33]. Across the surveys, data are collected from mothers and carers of children under the age of 5; malaria module questions pertain to service use for the last episode of fever and/or cough within a two weeks recall [31]. While there is a high degree of comparability between the survey instruments, the data sources differ in scope and coverage resulting in some differences in estimates [34]. Of the two the DHS is a more comprehensive resource, with the primary purpose of producing monitoring and impact evaluation indicators at national and regional level; these data allow for a consistent definition of a broader set of indicators considered in this analysis thus we opt for DHS over MICS estimates. Survey data for more recent MICS (round 4, corresponding to data collection years 2010–2011) were available only for a subset of counties at the time of the analysis with most country data presented in a summary report form. In the latter definitions of coverage indicators are narrower than nose adopted in our model with health seeking defined as a proportion of children under five years of age with fever receiving antimalarial medicines. Using the reports to fill in gaps in DHS data will result in lower estimates of coverage; we turned to these data only when no other data were available and used more dated DHS surveys if coverage estimates were higher than those reported in more recent MICS.

We assume health seeking and choice of care provider for malaria related fever is the same across febrile conditions; thus access to malaria case management and source of treatment assessed as a proportion of all-cause fever cases is deemed representative of malaria fevers.

We ignored recommendations to use parasitological diagnoses in estimating compliance with the recommended first-line therapy defined according to country policy [4]. Instead, we proxy malaria etiology with presumptive diagnosis indicated by prescription of an antimalarial medication, assuming that fevers treated only for other etiologies were not malaria. Malaria accounts for a variable share of febrile cases across countries depending on the transmission level [35]. Using presumptive diagnosis is supported by evidence from field trials and national surveillance that show that despite changes in policy to introduce diagnostic tests, symptomatic treatment remains the norm in most of Africa [19,22,27,36]. An additional argument supporting omission of diagnostic coverage in this model is presented by a recent evaluation of recall bias in DHS/MICS surveys pointing to a particularly poor recall of diagnosis and testing related to malaria episodes, suggesting the recall data substantially understate both the use of diagnostics and positive test results [37].

Compliance with the recommended first-line treatment is likely one of the most time sensitive indicators in our model, with the change toward ACTs representing a fairly recent intervention with increasing and differential rates of scale-up across countries. Data on compliance came from 2010–2012 surveys and where not available were filled in with information from the literature and WMR. To our knowledge there have been no comprehensive studies summarizing evidence on ACT implementation and scale-up across the region, in the absence of which country 2010–2012 trends on ACT deliveries to public and private sectors and ACT treatment courses distributed by the National Malaria Control Programs from WMR[38] could be used to gauge changes in ACT coverage over the period. The report shows that there was a large increase in the number of ACTs delivered from 2010 to 2011 in the region primarily through the Affordable Medicines Facility-malaria(AMFm) initiative subsiding ACTs for private and public sector distribution in Ghana, Kenya, Madagascar, Niger, Nigeria, Tanzania and Uganda. For most of these countries 2012 survey data were available; for Ghana, Kenya and Madagascar estimates were derived from 2010–2011 data that only partially capture these efforts. This is particularly an issue for Madagascar where the number of ACT treatments delivered increased nearly 7 fold in 2012 compared to 2011. The report also indicates that increased ACTs procurements from 2011 to 2012 reflect purchases for routine public sector deliveries, which increased by about 50% over the period while the funding through the AMFm facility declined slightly. Countries with the highest increase not captured by our data include Ethiopia, Mali, Nigeria, Rwanda, and Togo; to the extent that changes in number of ACTs delivered translate into higher coverage our estimates of effectiveness of malaria of case management are understated for these countries.

Limited country specific data are available on compliance levels by source of treatment. For a subset of countries for which these data were collected we estimate that on average 60% of treatments delivered in formal and about 40% in informal sectors comply with the country policy. We use the ratio to allocate country level compliance from the World Malaria Report [4] to formal and informal sectors where the parameter could not be estimated directly from the national surveys. To streamline the analysis, we proxy all treatments that are non-compliant with the official first-line therapy with Sulphadoxine-Pyrimethamine (SP).

For a subset of recent DHS surveys “drugs taken for fever/cough” sequence is followed with a sequence on length of drug administration (number of days (1–7)). Adherence with the ACT regimen is defined narrowly in our framework in terms of number of days an ACT drug was taken as reported in these surveys. Other dimensions for adherence to ACT drugs not captured with this definition could include appropriate dosage, timing of administration, and mode of administration (i.e. intake of fatty foods to facilitate absorption). Since adherence data from national surveys are sparse, often with unstable sample sizes due to low ACT uptake, we supplemented them with estimates from the research literature (S3 Table). Adherence was imputed for countries for which no information on the parameter could be identified by relating adherence data from the literature and the surveys to per capita health care expenditures. Studies of pharmacokinetics and pharmacodynamics of ALU showed that the last two doses (most likely to be missed by partially adherent patients) contribute most significantly to elevating lumefantrine concentrations to levels necessary to clear all parasitemia with incomplete regimens leading to increased risk of recrudescence [39,40]. We assume conservatively that patients that took an ACT drug for at least three days achieved an optimal drug concentration while those partially adherent remained parasitemic after the treatment.

While representative country level data are routinely collected on health seeking and type of treatment sought for fever data on other aspects of malaria case management in particular with respect to drug quality and antimalarial resistance are less ample. An indicator for drug quality is defined in the model as a proportion of antimalarial medications that are counterfeit or substandard and is populated with estimates from recent systematic reviews (S4 and S5 Tables)[41–45]. This literature however provides little insight on the quality of antimalarial medication distributed through public outlets; field studies that explored this aspect have shown that counterfeit and sub-optimal drugs are largely distributed through private and low level care providers. We thus assume that authentic formulations are dispensed through the formal facilities; counterfeit and suboptimal formulations are only factored in for treatments obtained from informal sector. Given limited geographic coverage of these data we applied common values across countries. Patient outcomes when treated with counterfeit or otherwise substandard medications are not well understood [26,27], partly because there are various reasons for failure in quality tests including absence of the API, incorrect API, incorrect dosage of API, and suboptimal release of API [26]. Due to lack of general guidance on relating drug quality to clearance of parasitemia, we assumed that treatment with drugs classified as substandard fails to clear parasitemia.

Antimalarial drug resistance is another key factor undermining efficacy of antimalarial therapy. Parasite resistance to artemisinins—the key compounds in ACTs—has been detected in the Greater Mekong sub-region but ACTs remain so far efficacious in Sub-Saharan Africa[4]. Consequently 0% resistance to first-line treatment is assumed for the countries sampled. Evidence on parasite drug resistance to non-ACT antimalarials is summarised in [46]. Some of these country estimates are based on trials conducted as far back as 2000; given the rate of resistance we instead applied a median value of parasitological failure rate of sensitive parasites for SP from the survey to all countries.

Evidence on clinical efficacy of first-line antimalarial medicines produced in controlled environments according to standard protocols were obtained from [4]. Mean failure rates at 28 days follow-up for ALU are about 1.96%; while for most countries failure rates are well below 5%, for Burkina Faso and Malawi the rate is 7%. Failure rates for AS+AQ formulations are on average about 3.91% with highest failure rates reported in Gabon (13.8%). Two countries in our sample recommend AS+SP as a first-line treatment for uncomplicated malaria; failure rates for the combination are reported at 0.5% and 2% for Somalia and North Sudan respectively.

Estimates of both effective coverage and systems effectiveness populated with survey data depend on the duration of the recall period. In particular, the probability of prompt (i.e. within one day) effective treatment (*E*_1_) defined as a target in the Roll Back Malaria Global Malaria Action Plan [47] is substantially lower than the effective coverage based 14-day recalls (*E*_14_) [48]. S2 Fig and S1 File provide the algorithm and national-level estimates of *E*_1_. Elsewhere in the paper the term effective coverage (*E*) refers to *E*_14_, in line with the widespread use and availability of survey data with 14 day recall periods.

## Results

### Malaria service performance indicators

**Fig 2:**
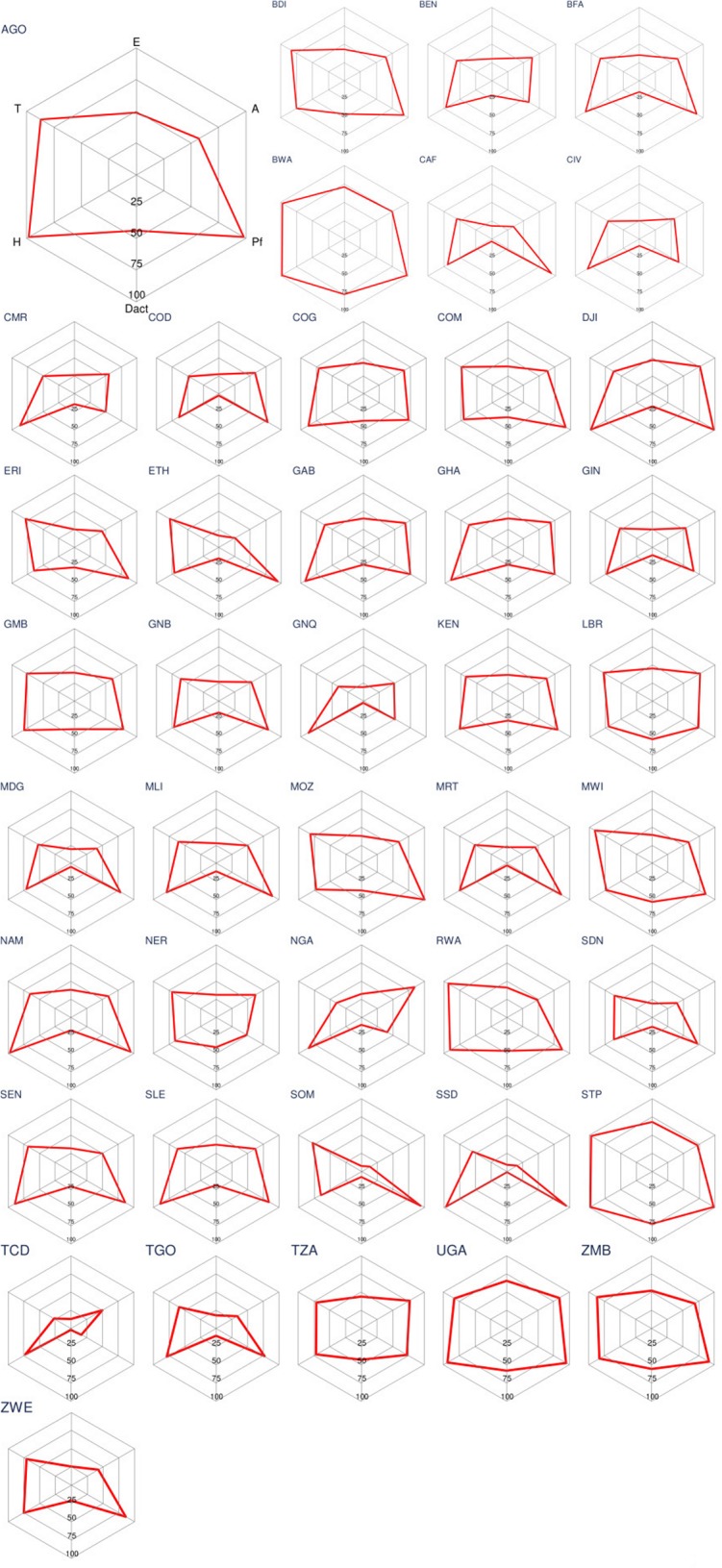
Effective Coverage and Malaria Service Performance Indicators by Country (%). Labeled on the axis are: *E* Effective coverage; *A* Access to any care provider; *Pf* Access to a formal care provider; *Dact* Compliance with the first-line antimalarial treatment; *H* Adherence; *T* Cure rate. Labels for country ISO3 codes are listed in the S1 Table.

Estimates of malaria service performance indicators for the 43 Sub-Saharan African countries are reported in S1 Table and are summarized graphically in Fig 2.

#### Access to malaria case management

Overall health seeking for malaria related fever is relatively high across the region; over half of fevers are treated in either formal or informal settings (median 59%, IQR 50%–84%). Access to malaria case management is lowest in Somalia, South Sudan, Ethiopia, Togo, and Central African Republic where less than a third of malaria cases receive any care. Uganda and Nigeria are the two countries with the highest reported level of treatment of about 84%. When treatment is sought, with a few exceptions, reliance on formal care providers is ubiquitously high across the region; about 80% of fever cases are treated in the formal medical sector (median 78%, IQR 50%–91%). An outlier is Chad where we estimate that only 17% of treatments are obtained from formal health care providers. Informal outlets are also the primary source of care for malaria related fevers in Nigeria and Niger: over half of fever cases for which care is sought are treated informally in these countries.

#### Provider compliance and patient adherence

Compliance with the recommended first-line medication for uncomplicated malaria remains low with less than a third of fevers treated according to the national guidelines (median 28%, IQR 12%–59%). Only a handful of countries achieved a near perfect compliance with the first-line therapy including Botswana, Malawi, Rwanda, Sao Tome and Principe, Somalia, and Zambia: about 90% of fevers are treated with the first-line antimalarial in these countries. When treated with an ACT we find that patients generally adhered with the drug regimen; over 80% of patients completed the recommended 3-day course (median 81%, IQR 71%–84%).

#### Drug quality and resistance

Based on the literature review we estimate that on average 17% of ALU purchased from pharmacies, shops, or other informal sources are of substandard or poor quality with a range between 0 and 62%. A much higher prevalence of counterfeits is estimated for SP with a mean of 33% and a range between 0 to 66%. While ACTs remain effective within the Sub-Saharan Africa, resistance to non-ACT antimalarials is estimated within the 20 to 69% range across the region; when estimating effective coverage we took the midpoint and assumed that on average SP treatment fails in 40% of cases.

#### Cure rate

The overall cure rate of antimalarial drugs is estimated at about 66% (median 65%, IQR 53%-80%). Given the paucity of data on the key parameters that contribute to estimation of the cure rate, the variation between countries is driven by the share of treatments obtained from formal care providers. Whereas ACT resistance is not yet important in Africa, fevers treated in informal setting including markets, shops, and relatives are less likely to receive the official first-line antimalarial and more likely to receive antimalarial medication that is counterfeit or otherwise substandard medication. We estimate average antimalarial drug cure rate at or below 60% for malaria fevers treated in Chad, Democratic Republic of the Congo, Equatorial Guinea, Mauritania, South Sudan, and Central African Republic.

### Overall effective coverage and system effectiveness

**Fig 3:**
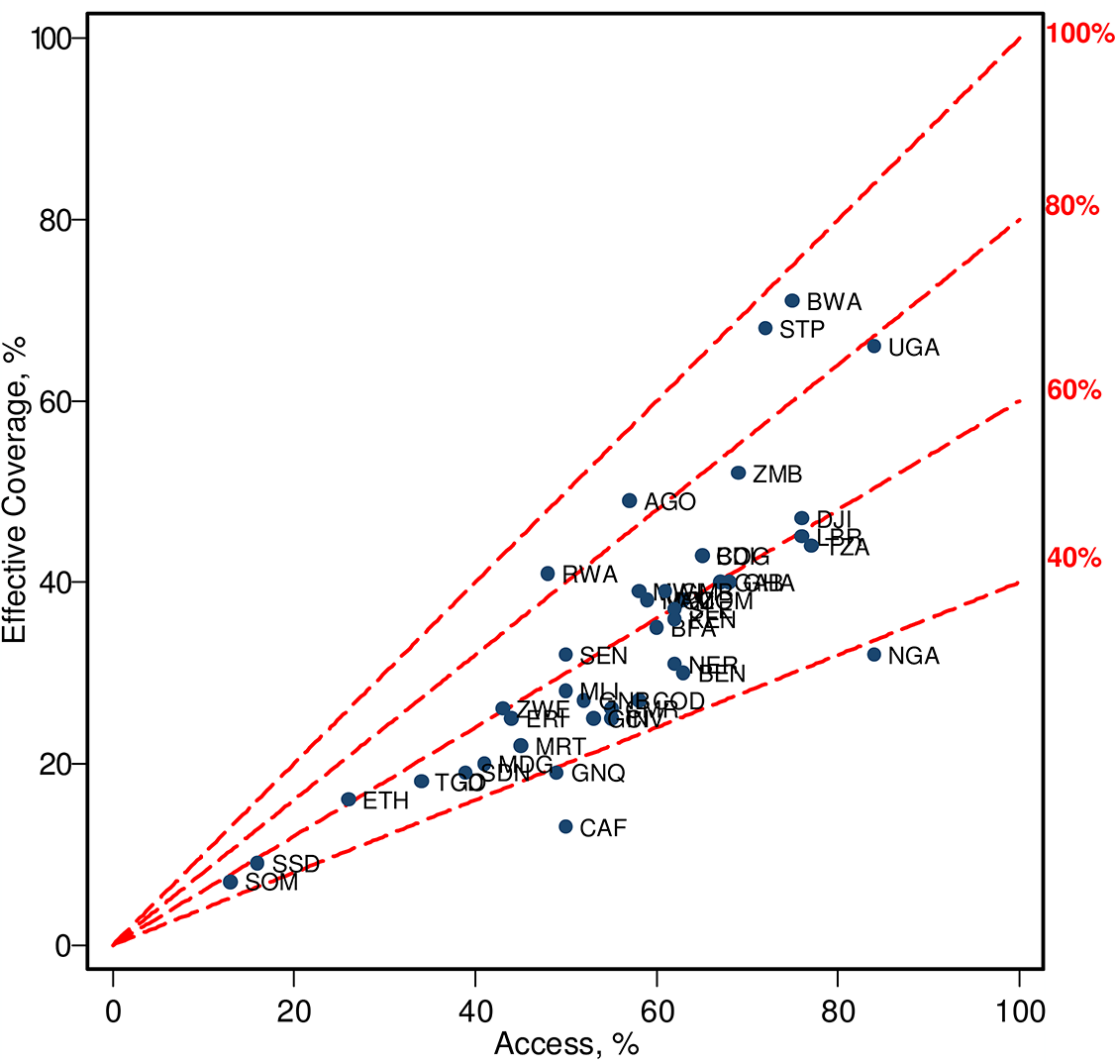
Effective Coverage (*E*) and Access to Any Provider (*A*) by Country (%). Dashed lines starting with a 45 degree angle and steeper illustrate the ratio of fever episodes treated effectively (Effective Coverage) over episodes for which treatment was sought (Access). Labels for country ISO3 codes are listed in the S1 Table.

Effective coverage of malaria case management varies from 7% in Somalia to as high as 71% in Botswana; only about 40% of fevers are managed effectively in the region (Fig 3; S1 Table). Effective coverage is particularly low in a cluster of countries in the North Eastern part of the continent including Somalia, South Sudan, Chad, Ethiopia, Central African Republic and North and South Sudan; slightly over 10% of fever cases are treated effectively in these countries. Focusing on the performance of formal care providers we find that less than 6% of malaria fevers are managed according to the national guidelines in the region. Systems effectiveness ranges from less than 1% in Chad and South Sudan to at most 60% in Botswana, Sao Tome and Principe, and Uganda.

Fig 3 illustrates the large gap between access to malaria case management (access) and cure with the treatment obtained (effective coverage). While on average 60% of fever cases seek treatment, only a third clear parasitemia; that is about half of the potential gain from treatment is lost to inefficiencies. By far the most dramatic difference between access and treatment outcomes is found in Nigeria where 84% of malaria fevers accessed a care provider; however less than 32% of malaria fevers were cured in this country. As a percent of fevers treated, effective coverage is lowest in Chad where less than a third of treatments are effective; a similarly low effectiveness of malaria case management is estimated for Nigeria, Equatorial Guinea, Cote d’Ivoire, the Democratic Republic of the Congo, Guinea, Cameroon, Benin, North Sudan, Madagascar, and Mauritania where less than half of the treatments result in a cure. We find the highest effective coverage in Botswana, Sao Tome and Principe, Angola, Rwanda, Uganda, and Zambia with over three fourths of all malaria related fevers treatments effective.

Based on the framework adopted in this analysis effective coverage is necessarily higher than system effectiveness (S3 Fig), because the former includes the contribution of informal providers, while the latter represents the effectiveness of the formal health system only. In general, the difference between the metrics is less in countries with high effectiveness (in particular Botswana) than in countries where effectiveness is lower. This difference is clearly driven by the data and assumptions that imply that treatment provided by the informal system is of worse quality on several of the indicators. An important example is Nigeria, for which we estimate relatively high access, but poor compliance, associated with a low proportion of patients accessing formal facilities.

### Impact of performance indicators on the level of effective coverage

Summarized in a set of radar plots for each of the 43 Sub-Saharan African countries indicators of case management performance allow for identification of bottlenecks in service provision along the inputs surveyed. Inefficiencies in service provision appear as a shift on the operation curve toward the center of the diagram (away from 100% coverage toward 0% at the center of the diagram) and imply that for a significant proportion of target population the service is failing to meet the standard of care. For instance in Angola access is the lowest coverage indicator; once care is sought, however, patients are generally treated appropriately in this country. In Djibouti access to the first-line antimalarial therapy is the key bottleneck in service provision. Operational curves for Benin, Cameroon, Central African Republic, and Chad among other reveal failures in malaria service delivery along multiple indicators including access, treatment seeking in formal sector, first-line antimalarial, and drug efficacy.

**Fig 4:**
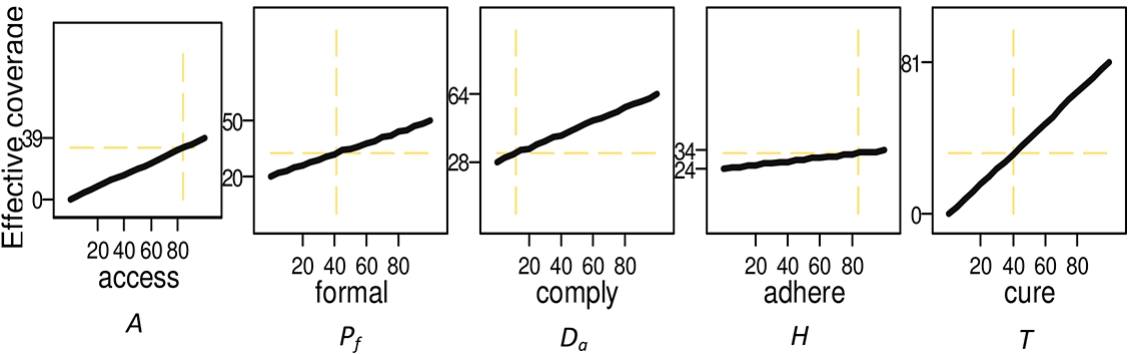
Impact of Service Parameters on Level of Effective Coverage: Nigeria. Labeled on the x-axis are: A Access to any care provider; Pf Access to a formal care provider; Da Compliance with the first-line antimalarial treatment; H Adherence; T Cure rate. Effective coverage. E, evaluated by varying each of the service indicators from 0 to 100% while holding all other inputs at base value. Values of effective coverage at lowest and highest values of inputs are labeled on the y-axis to illustrate the sensitivity. Baseline values of inputs and effective coverage are shown in dash lines.

Overall the drivers of losses in effectiveness are strongly context specific. The impact of different factors can be disentangled by evaluating a series of counterfactual scenarios where the change in effective coverage is assessed by varying one of the parameters while holding all other inputs at their baseline values. In Fig 4 we disaggregate the impact of constraints on the overall effectiveness of treatment using as an example a high malaria burden country of Nigeria. The outcome of the model- effective coverage- is plotted on the y-axes, labels indicate the estimated level of effective coverage at lowest and highest values of the parameters (0% and 100%); dash lines show the level of effective coverage and respective input values at the baseline.

Over 80% of fever cases seek care in Nigeria; yet at such high access to care the effectiveness of treatment delivered is low- only about a third of treatments result in a cure. Expanding access further without addressing inefficiencies in the delivery to 100% of episodes will only increase effective coverage to about 46%. Another dimension- source of treatment- constraint by access would only raise effective treatment to half of the cases for which care is sought if all treatments are delivered through the government facilities where presumably high quality medication is administered by qualified staff based on clinical assessment and providing instruction on the appropriate drug regimen. Compliance with the recommended first-line antimalarial is particularly low in Nigeria, it is one of the primary reasons for low levels of effective treatment in the country; about one tenth of episodes for which care is sought are treated according to the national guidelines. Focusing policy efforts on compliance will improve effectiveness of malaria case management at the highest rate than changing any other parameter considered here; however even at 100% compliance without improvements in other areas significant losses to inefficiencies in services delivery will be incurred with only about 63% of fevers treated effectively.

**Fig 5:**
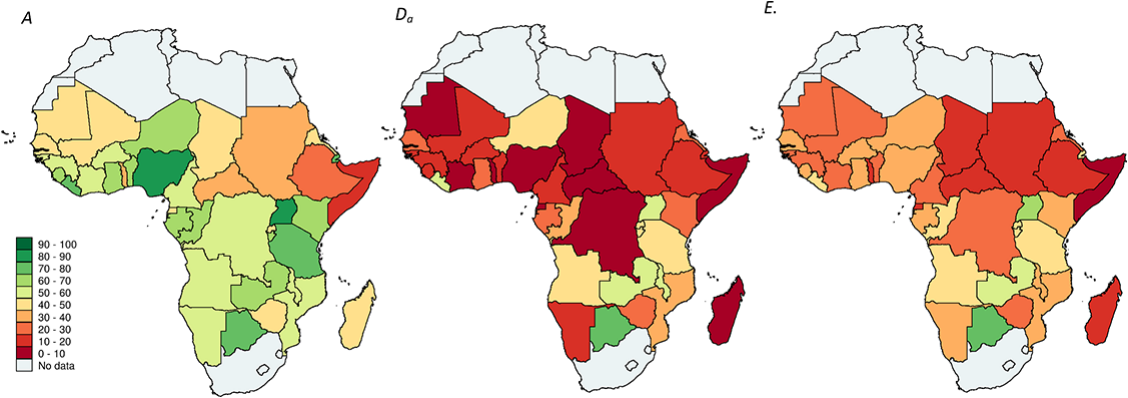
Access, First-Line Antimalarial Treatment, and Effective Coverage of Malaria Case Management by Country. A. Access to any provider; *D*_*a*_ Compliance with the first-line antimalarial treatment; C. Effective coverage. Maps were generated by authors using *spmap* command[73] in Stata 13 SE; Country coordinates were sourced from Global Administrative Areas (http://gadm.org).

Another way to illustrate this point is by comparing the three sets of maps with color gradients used to show country levels of access to health services (Fig 5(*A*)), compliance with the first-line treatment (Fig 5(D_a_)), and effective coverage (Fig 5(E)). For the most part effective coverage appears to follow closely patterns in compliance; high levels of access are offset by treatment with less efficacious antimalarial regimens. Yet, across countries with low compliance the level of effective treatment is markedly higher in settings where patients seek care and rely more heavily on formal care providers.

## Discussion

Despite the availability of efficacious drugs, the failure to treat malaria fevers effectively probably contributes much to the still unacceptably high burden of the disease. We estimate country-level values ranging from 8% to 72% for the effective coverage and from 0.1% to 68% for systems effectiveness. Effective coverage appears to be particularly low in a cluster of countries in the central and North Eastern part of the continent including Somalia, South Sudan, Chad, Ethiopia, Central African Republic and North and South Sudan. Effective treatment of malaria depends on successful completion of each step in the service delivery path. Failure at any step means that the infection will be inadequately treated or not treated at all, so all steps in the chain need to be high if effective coverage is to achieve an acceptable level. Our analysis suggests that there are issues needing to be addressed at each step, but the country level data are very uncertain. This is mainly because of a lack of country level data on key indicators including adherence, quality and resistance of non-ACT antimalarials, on which drugs are used by care providers, and on the ways in which the available data relate to patient outcomes.

Nationally representative DHS and MICS are the key sources of data on coverage and utilization of malaria control services in endemic countries. While these surveys provide extensive information on access to care and other coverage indicators, the data collection rounds are widely spaced; with an average frequency of every 5 years for DHS and 3 years for MICS per country. In our analysis for about half of the sample, data came from 2011–2012 (S2 Table), for these countries consistent estimates of access, source of care, compliance and adherence could be estimated from the DHS. For the remaining countries with less recent DHS data we made every effort to bring these estimates into the same time horizon by drawing on 2010–2011 MICS and 2012 WMR, albeit at the cost of potentially introducing uncertainty by combining estimates from different sources with somewhat varying sampling frame, quality, and scope.

The level of uncertainty in estimates of effective coverage induced by these data limitations differs by indicator. The implications for interpretation are two-fold. First the quality of estimates varies by country with more uncertainty around estimates for countries with no recent DHS surveys. Second these methodological differences may bias comparisons of coverage indicators across settings. To gauge the magnitude of the problem we summarized coverage indicators for a subset of countries with consistent DHS 2011–2012 and found the range and the median effective coverage estimates for this subset to be nearly identical to that estimated over the full set of countries; this suggest that while estimates are imprecise for a given setting, differences in estimated effectiveness across the region do reflect underlying inefficiencies in service delivery rather than data inconsistencies

Field studies of adherence [49–51] are generally not designed to obtain data representative of the normal behaviour of the population. Patients may be informed in advance of possible follow-up visits[52–54]; ACTs are often given free of charge after a confirmed malaria diagnosis, with counselling by specifically trained staff on administering treatment (dosage, drug schedule, intake with fatty foods, re-administration of doses) [55,56]. We did not explicitly consider accuracy of dosing, since analyses that assessed treatment outcomes among patients that received suboptimal dosage of antimalarial medication found clearance rates similar to those fully adherent with respect to dosage [57]. Field studies have shown dosing accuracy of about 80–99% at formal facilities [58–61] and 65–80% accuracy at retail outlets [49,57,59,62,63] with rates differing by type of antimalarial, formulation, and packaging.

There is abundant recent evidence on the incidence and pervasiveness of counterfeit and substandard antimalarial medication in the endemic region, so we include drug quality as a separate indicator in our framework. However there is a clear lack of representative country level data to inform this parameter. Generalizing from local studies poses a number of difficulties: differences in study design, testing tools, and thresholds against which drug samples are evaluated further obscure the interpretation, making these estimates of impact on effectiveness very uncertain [26,27,64]. The inference about the population risk is particularly problematic as the surveys of drug quality are very often based on convenience samples of providers and do not relate directly to consumption. Because of the challenge of inferring national level values from such surveys we assumed one value for drug quality for all 43 countries; by doing so we failed to capture the extent of variation across the region as suggested by the limited field data. Furthermore, patient outcomes when treated with counterfeit or otherwise substandard medications are not well understood. Assuming conservatively that poor quality treatments always fail might bias our estimates of effective coverage.

Despite the difficulty of working with these sparse data and hence borrowing of information across countries (which tends to average out the differences), we estimate that there is a substantial variation between countries in compliance, drug quality, and adherence. A consequence is that the relative importance of these factors also varies between countries. In contrast, ACT resistance is currently not a significant contributor to loss of systems effectiveness while resistance to other drugs is an important contributor in the informal sector. Again, our estimates probably understate the variation between countries, because we lack a comprehensive source of accurate and up to date information on resistance to the drugs that are actually in use. We adopted one threshold value, based on an average level of drug resistance for SP, for all countries. This does not accurately reflect variation in the level of resistance across settings because a range of antimalarial therapies aside from SP and the first-line antimalarial drugs are in circulation. These include artemisinin monotherapies which are likely much more effective than SP, as well as chloroquine, to which resistance is widespread.

Further the model for service delivery relies on a number of generalizations to assess patient outcomes along the malaria case management delivery path, something we have addressed above by indicator. Given the quality of data our focus here is to present a measure of effective coverage that could be easily interpreted and estimated with available data rather than to precisely parameterize clearance rates for malaria case management per se. We effectively ignore heterogeneity in response to treatment that stems from malaria age-incidence particular to a given setting, levels of exposure and resistance to antimalarial regimens available in a given country, the range of antimalarials used, quality of drugs and its distribution across vendors, response to incomplete drug schedule among other. Constrained by data availability and evidence by imposing linearity in combining coverage indicators to measures of effectiveness of case management we likely understate the level of clearance achieved with routine care.

In the context of these concerns about data quality especially at country level, it would be premature to use these estimates to pinpoint specific national systems failings, because so many of the data are unreliable. We present our findings as point estimates without properly representing the uncertainty around these parameters, because many of the values depend on assumptions about generalizability that we cannot test. A conclusion is that there is an urgent need for improved data on a key essential set of these indicators, especially on provider compliance, adherence and drug quality. Forthcoming work by [65] overcomes some of these limitations relying on econometric and statistical modelling to yield estimates of ACT coverage and malaria diagnostics for the region. Further on-going efforts on collecting and standardizing data on quality of antimalarial medication and drug resistance led by WWARN will help inform more reliably these key indicators for the region [66,67].

The counterfactual analysis indicates the potential improvement in effective coverage that could be achieved by improvements in each of these factors, illustrating the constraints that the downstream factors impose on treatment outcomes. The impact of any given parameter on effectiveness is scaled by the level other indicators. Compliance, adherence, and cure rates are more important in settings where access is high. Similarly, in the presence of alternate less effective treatments adherence and quality of first-line drugs will only have a major impact where provider compliance is relatively good. For the same underlying statistical reason, within-country heterogeneities (including local inequity in any of the parameters) will also tend to reduce effectiveness below the estimates we obtain by assigning single national-level values for each parameter.

In terms of the effects of health systems interventions, expanding access to treatment translates into higher levels of effective coverage in settings where other service indicators perform well; switching to a more efficacious treatment regimen can only improve effective coverage if treatment is sought and the drug is taken. In general policy efforts targeting marginal improvements across multiple dimensions of service provision will result in a greater improvement in effectiveness than interventions focused on scaling up only one particular aspect of malaria case management. It follows that analysis of the potential public health impact of improving each parameter contributing to efficacy needs to consider the values of the other parameters. Such analysis would also need to consider the impact on the pool of infectious hosts contributing to malaria transmission, taking into account whether treatment is prompt, which affects burden both because it modifies the chances of onward transmission, but also because delayed treatment increases the risk that an uncomplicated episode progresses to a more severe or a fatal one and[6]. As a consequence of these effects, treatment effectiveness is not only of public health importance in its own right, but it also modifies the impacts that are achieved by preventive interventions. Effective case management thus needs to be considered as part of an overall integrated control and elimination strategy, and the potential impacts of both preventive and curative interventions should be evaluated within an inclusive framework that considers both, as well as their interactions. Naïve analyses might suggest that different interventions represent alternative ways of averting the same disease burden, but simulations suggest that the impact of some preventive interventions (in particular, long-lasting insecticide nets) could correlate positively with the quality of curative care [68].

Our analysis does not identify the specific factors directly responsible for low effectiveness and hence do not suggest specific strategies to address inefficiencies in service delivery. Low levels of treatment seeking might imply poor allocation and deployment of resources and facilities or reflect population perception of severity of illness or quality of care [16,17,69]. Incidence of non-compliant treatment on the other hand might point toward problems with governance and regulatory efforts, the supply system or affordability of the ACT therapy [16,69]. Further incorrect administration of effective drugs could indicate gaps in knowledge of care providers and caretakers with respect to appropriate regimen, failure of care providers to communicate instructions correctly or again point to affordability of treatment [19,49]. Quality of antimalarial compounds is another complex dimension that signals broader systems problems on the legal and enforcement side as well as poor infrastructure and failure to enable proper storage and handling of medical supplies in endemic settings [26,27,64]. However, the weak correlation of effective coverage with indices of economic development indicates that resource constraints are only a small part of the story (S1 Fig). Some countries with relatively strong economies including Equatorial Guinea, Gabon, Namibia, Ghana, and North Sudan have low success rates of treatment for patients that seek care for malaria, even in comparison with much poorer countries.

In addition to the need for timely and quality data at country level, there will be a need for improvements to our algorithm to account for non-linearity in metrics and increasing use of diagnostic tests. Symptomatic treatment remains the norm in most of Africa [22,36,70] and we implicitly assume care seeking and treatment are independent of the underlying cause of fever. Increasingly though, malaria treatments are based on parasitological diagnoses and health facilities surveys found 85% (in Ghana) and 84% (in Benin) of prescriptions to be based on positive test results[71]. Results from Senegal are similar[72]. Treatment conditional on diagnosis ensures antimalarials are administered only to patients with the infection. This is particularly relevant with reductions in transmission which mean that presumptive treatment leads to massive over-prescription of costly ACT therapy. Incorporation of parasitological diagnosis into the algorithm would lead to lower estimates of provider compliance with official guidelines, but should have little effect on estimated effective coverage, since malaria diagnosis mainly improves management of non-malaria fevers.

Overall, our study has highlighted striking gaps in the availability of fundamental data on the performance of health systems in sub-Saharan Africa, with most of our estimates, except measures of access, depending on very partial unreliable data with low spatial and temporal resolution. Further analyses, considering temporal trends, sub-national heterogeneity in access, and geographical patterns of disease, will make it possible to compare between countries the potential public health impacts of improving the different indices, alongside the impacts of other interventions. Although the estimates we have made so far are crude, they already indicate in broad terms where additional data and improvements in systems performance are most needed.

## Supporting Information

S1 Fig Malaria Service Indicators (Log) Against EIR, GNI per Capita, Government Health Care Expenditures per Capita (PPP), and DTP3 Coverage by Country. Labelled on the y-axis are: *A*—access to any care provider; *D*_*a*_—compliance with the first-line antimalarial treatment; *H*—adherence with the drug regimen; *E14*—effective coverage.
(TIFF)

S2 Fig Calibration Curve Relating Estimates of Access within 24-Hours to Access During 14-Day Recall Periods. The horizontal axis gives the percentage of 14-day fever recalls in nationally representative surveys reporting accessing treatment. The red dots: survey data on the percentage of fevers accessing treatment within 24 hours; continuous black line: percentage of fevers accessing treatment within 24 hours as estimated by the approach of Crowell *et al* [48]; dashed black line: percentage of fever bouts (continuous series of days with fever) during which treatment was accessed as estimated by the approach of Crowell *et al* [48]; grey line: percentage of fevers accessing treatment promptly, if all access is within 24 hours.
(TIFF)

S3 Fig Relationship between Estimates of Systems Effectiveness (*S14*) and Effective Coverage (*E14*) for Malaria by Country (%). The diagonal line corresponds to the estimates that would be obtained for countries where the two quantities are equal. Labels for country ISO3 codes are listed in the S1 Table.
(TIFF)

S1 File An algorithm to convert a 14-day effective coverage (*E*_14_) metric to estimates of a 24 hour effective coverage (*E*_1_) measure. (DOCX)

S1 TableIndicators for Malaria Service Delivery and Effective Coverage by Country.
$E_{1}^{+}$ is the estimate of *E*_1_ obtained from survey data; $E_{1}^{*}$ is the estimate obtained from *E*_14_ using the calibration curve; $E_{1}^{**}$, the proposed best estimate for each country, is the value of $E_{1}^{+}$ if this exists, and otherwise the value of $E_{1}^{*}$; ^a^ weighted with population at risk from [38].(DOCX)

S2 Table Data Sources by Parameter and Country. ^1^ Access of 32% is estimated from the MICS(2010); ^2^ Access of 39% is estimated from the MICS(2010); 3 Access of 13% is estimated from the MICS(2008); ^4^ Access of 30% is estimated from the MICS(2010); ^5^ Access of 53% is estimated from the MICS(2010); ^6^ Access of 59.5% is estimated from the MIS(2010); ^7^ Access of 36.9% is estimated from the MIS(2012). ^8^ Most estimates are derived from the DHS surveys, and represent proportion of treated fever cases that sought treatment from formal medical care providers including hospitals, health centers, health posts, and community health workers. For countries where DHS surveys were not collected estimates were imputed. The imputation relied on identifying a country with non-missing access data with similar levels of transmission, DTP3 coverage, access to care for fever, per capita health care spending, number of nurses and midwives, percent rural, and population density. Countries were first ordered by level of transmission (low (<10) and high), and then consecutively by each of the other factors, removing most dis-similar records. We weighted stronger similarities in DTP3, access and spending, then the remaining 3 factors. ^9^ Adherence was imputed for countries for which no information on the parameter could be identified by relating adherence data from the literature and the surveys to per capita health care expenditures. Evidence from the literature suggested uniformly high adherence to ALU across settings. ^10^ Evidence on parasite drug resistance to non-ACT antimalarials is summarised in [46]. Some of these country estimates are based on trials conducted as far back as 2000; given the rate of resistance we instead applied a median value of parasitological failure rate of sensitive parasites for SP from the survey to all countries. ^11^ Given the limited geographic coverage of the data a median value over the estimates extracted from the literature were applied to all countries.
(DOCX)

S3 Table Data Sources and Point Estimates of Adherence with the First-Line Drug Regimen. (DOCX)

S4 Table Data Sources and Point Estimates of Drug Quality: ALU. (DOCX)

S5 Table Data Sources and Point Estimates of Drug Quality: SP. (DOCX)
