# Supplementary figures and images for: Effective Coverage and Systems Effectiveness for Malaria Case Management in Sub-Saharan African Countries
Source: PLoS One. 2015 May 22;10(5):e0127818. doi: 10.1371/journal.pone.0127818 (PMC4441512; doi:10.1371/journal.pone.0127818)

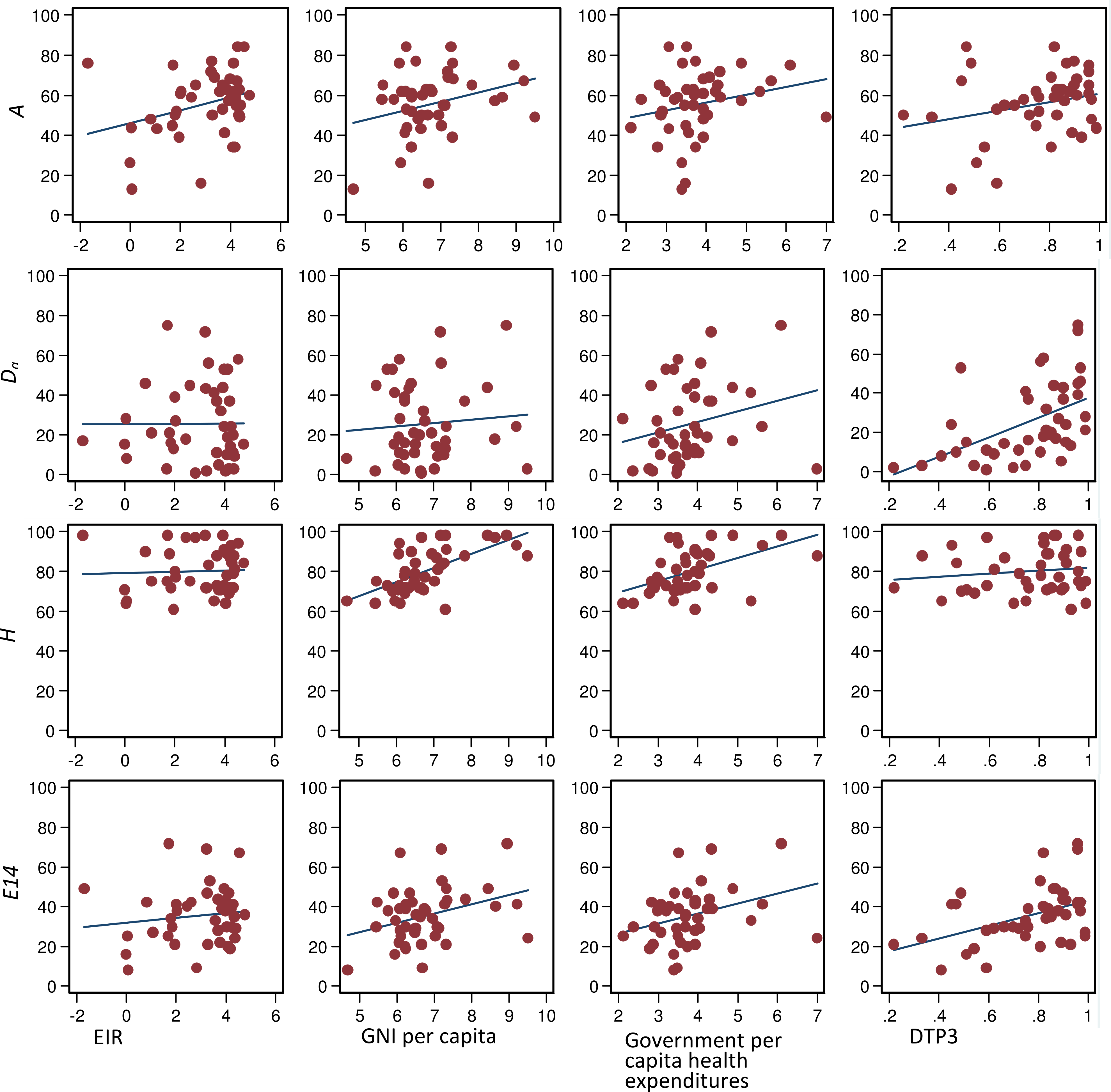

Supplement: S1 Fig — Labelled on the y-axis are: A—access to any care provider; D a—compliance with the first-line antimalarial treatment; H—adherence with the drug regimen; E14—effective coverage. (TIFF) [file pone.0127818.s001.tiff]

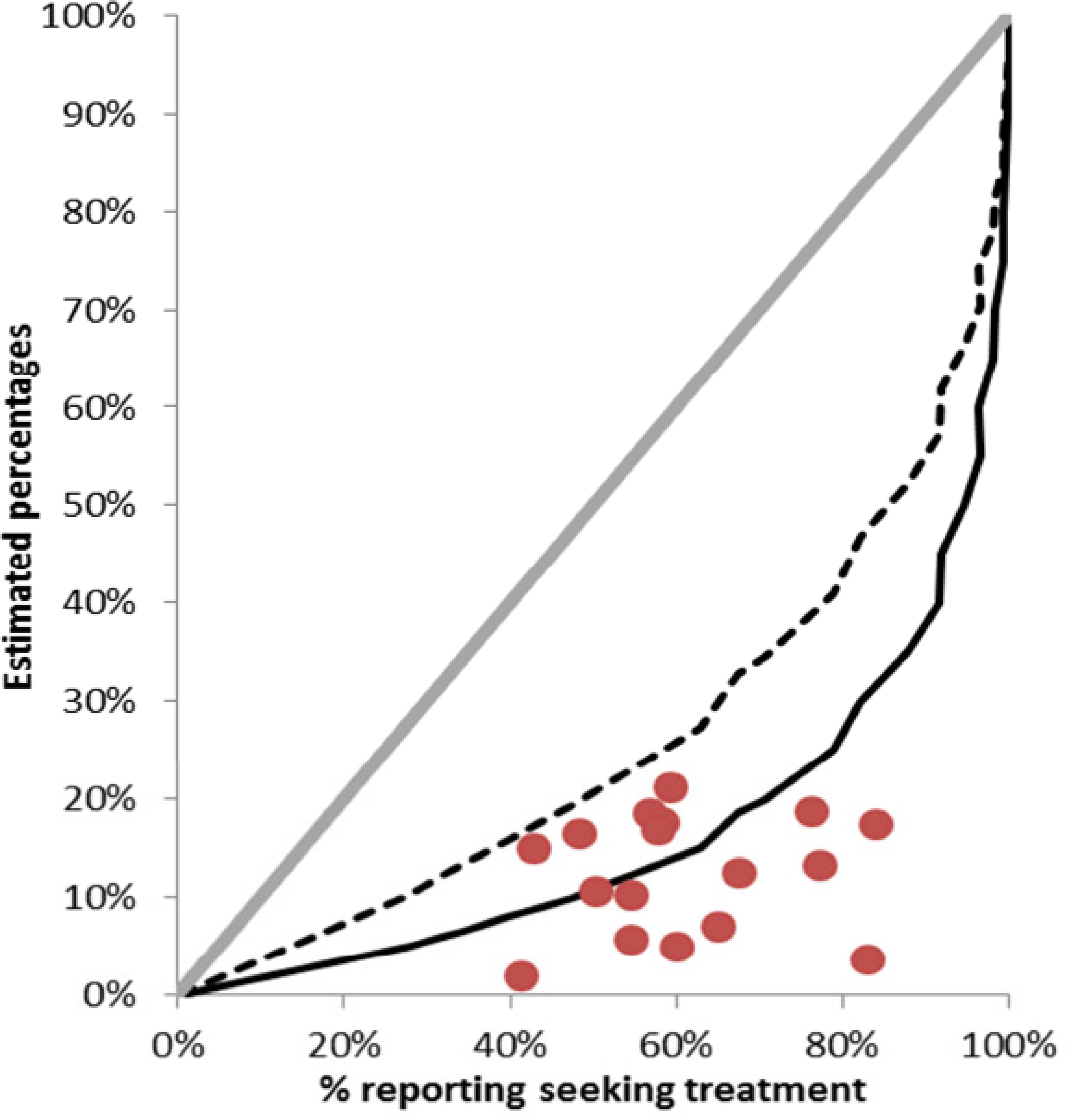

Supplement: S2 Fig — The horizontal axis gives the percentage of 14-day fever recalls in nationally representative surveys reporting accessing treatment. The red dots: survey data on the percentage of fevers accessing treatment within 24 hours; continuous black line: percentage of fevers accessing treatment within 24 hours as estimated by the approach of Crowell et al [48]; dashed black line: percentage of fever bouts (continuous series of days with fever) during which treatment was accessed as estimated by the approach of Crowell et al [48]; grey line: percentage of fevers accessing treatment promptly, if all access is within 24 hours. (TIFF) [file pone.0127818.s002.tiff]

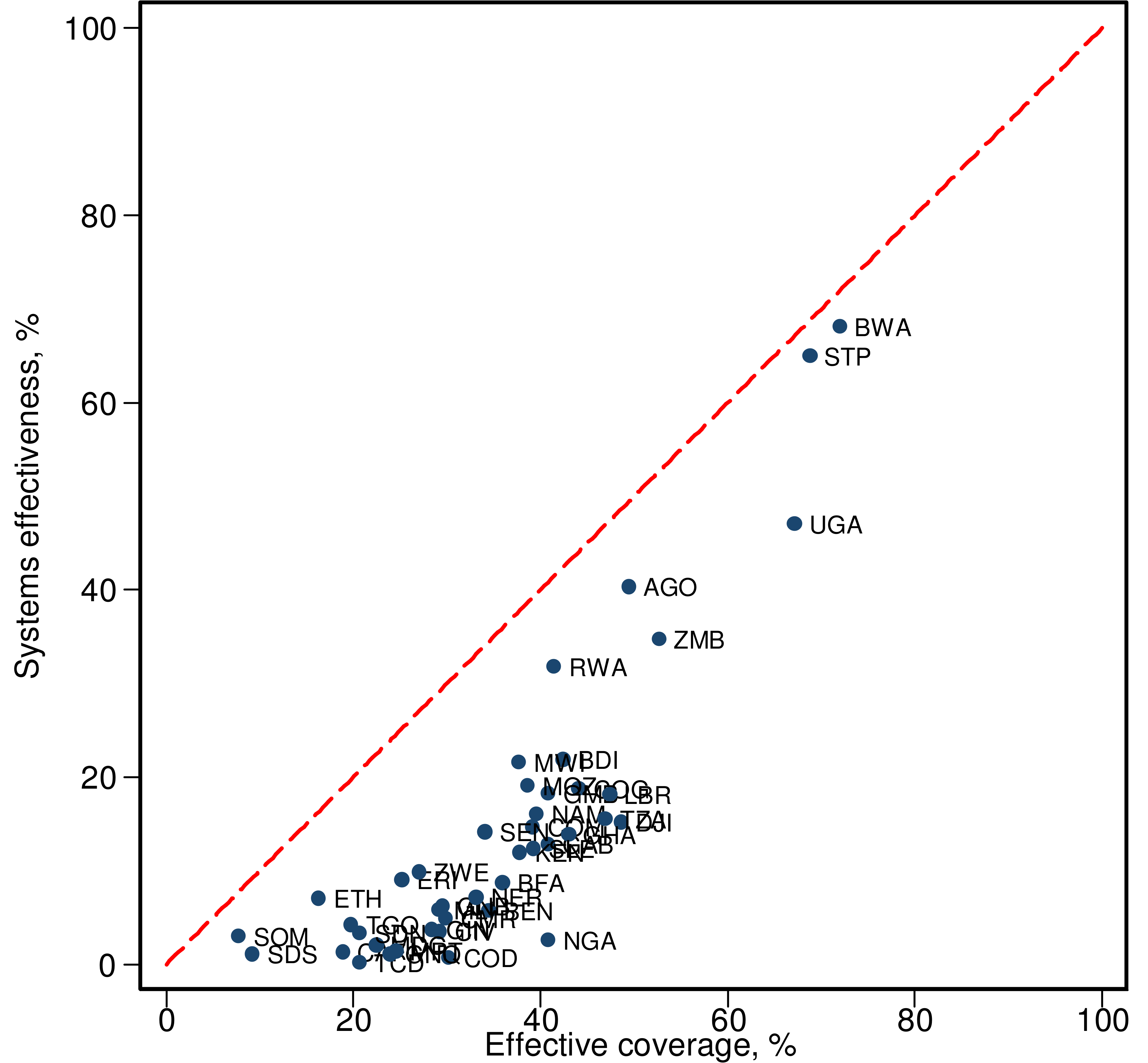

Supplement: S3 Fig — The diagonal line corresponds to the estimates that would be obtained for countries where the two quantities are equal. Labels for country ISO3 codes are listed in the S1 Table. (TIFF) [file pone.0127818.s003.tiff]
