# Supplementary material for: Effective Coverage and Systems Effectiveness for Malaria Case Management in Sub-Saharan African Countries
Source: PLoS One. 2015 May 22;10(5):e0127818. doi: 10.1371/journal.pone.0127818 (PMC4441512; doi:10.1371/journal.pone.0127818)
Supplement: S1 File — (DOCX) [file pone.0127818.s004.docx]

The proportion of patients accessing treatment within 24 hours (prompt treatment), were available for 17 countries (S1Table). For the other countries a previously published mapping of 14-day recalls of receiving treatment onto the percentage receiving prompt treatment [1] was used to estimate the proportion of fevers accessing treatment within 1 day *A*_1_, from the 14-day recalls (*A*) (S2 Fig.). This approach treats the probability that treatment is sought on a given day of illness as independent of how long the child has been ill, making use of the data on the natural history of *P. falciparum* in malaria therapy patients to approximate the temporal patterns of fever in untreated patients. For most of the countries for with field data on prompt access there is reasonably good agreement between the two measures but Nigeria is anomalous in having high access in the 14-day recalls, but very low prompt access to care.

Effective coverage within 24 hours was estimated by assuming proportionality to access, i.e.$E1=\frac{E14A1}{A}$: the same constant of proportionality was used to obtain estimates of systems effectiveness within 24 hours (S1Table). Estimates of the percentage of fever bouts (uninterrupted series of days with fever) that receive treatment were also made using the same approach (S1Table), making use of the distributions of durations of fever bouts in the malaria therapy patients[1].

Reference List

1. Crowell V, Yukich J, Briet OJ, Ross A, Smith T (2013) A novel approach for measuring the burden of uncomplicated Plasmodium falciparum malaria: application to data from Zambia. PLoS ONE .
