# Supplementary material for: Effective Coverage and Systems Effectiveness for Malaria Case Management in Sub-Saharan African Countries
Source: PLoS One. 2015 May 22;10(5):e0127818. doi: 10.1371/journal.pone.0127818 (PMC4441512; doi:10.1371/journal.pone.0127818)
Supplement: S1 Table — E1+ is the estimate of E 1 obtained from survey data; E1* is the estimate obtained from E 14 using the calibration curve; E1**, the proposed best estimate for each country, is the value of E1+ if this exists, and otherwise the value of E1*; a weighted with population at risk from [38]. (DOCX) [file pone.0127818.s005.docx]

| ISO3 country code | Country | Access to any provider | Access within 24 hours | Access to formal care provider | Compliance | Adherence | Cure rate | | Effective coverage | | | | | Systems effectiveness | | |  |
| --- | --- | --- | --- | --- | --- | --- | --- | --- | --- | --- | --- | --- | --- | --- | --- | --- | --- |
|  |  |  |  |  |  |  | ACT | SP | 14-day | 1 day | 1 day | 1 day | per bout | 14 day | 1 day | per bout |  |
|  |  | *A* | *A_1_* | *P_f_* | *D_a_* | *H_a_* | *T_a_* | *T_o_* | *E* | $E_{1}^{+}$ | $E_{1}^{*}$ | $E_{1}^{**}$ | *E_b_* | *S* | *S_1_* | *S_b_* |  |
| AGO | Angola | 56.9 | 18.5 | 98 | 77.1 | 97.6 | 98.5 | 55.4 | 49.5 | 16.1 | 8.9 | 16.1 | 18.3 | 40.4 | 8.2 | 16.2 |  |
| BEN | Benin | 63.4 |  | 58.2 | 32.0 | 72.4 | 91.9 | 48.1 | 34.5 |  | 6.2 | 6.2 | 12.8 | 5.7 | 1.0 | 2.1 |  |
| BWA | Botswana | 75.1 |  | 97.5 | 99.2 | 97.5 | 98.5 | 55.3 | 72.0 |  | 12.9 | 12.9 | 26.7 | 68.1 | 19.0 | 33.1 |  |
| BFA | Burkina Faso | 60.1 | 4.9 | 90.5 | 24.7 | 83.9 | 91.5 | 54.1 | 35.9 | 2.9 | 6.4 | 2.9 | 13.3 | 8.7 | 1.6 | 3.2 |  |
| BDI | Burundi | 65 | 17.5 | 99.9 | 69.6 | 74.5 | 93.6 | 54.5 | 42.4 | 11.7 | 7.6 | 11.5 | 15.7 | 21.8 | 3.9 | 8.1 |  |
| CMR | Cameroon | 54.5 | 5.7 | 50.4 | 26.1 | 86.8 | 88.2 | 46.7 | 29.9 | 3.3 | 5.4 | 3.1 | 11.1 | 4.8 | 0.9 | 1.8 |  |
| CAF | CAR | 33.9 |  | 93.3 | 9.0 | 69.1 | 97.8 | 54.6 | 18.9 |  | 3.4 | 3.4 | 7.0 | 1.3 | 0.2 | 0.5 |  |
| TCD | Chad | 50.1 |  | 17.4 | 3.0 | 72.4 | 85.0 | 40.6 | 20.7 |  | 3.7 | 3.7 | 7.7 | 0.1 | 0.0 | 0.0 |  |
| COM | Comoros | 63 |  | 91.8 | 51.5 | 71.0 | 97.5 | 54.3 | 39.2 |  | 7.0 | 7.0 | 14.5 | 14.7 | 2.6 | 5.4 |  |
| COG | Congo | 61.4 | 7.0 | 74.2 | 57.0 | 88.3 | 89.8 | 50.6 | 44.1 | 5.0 | 7.9 | 4.8 | 16.3 | 18.7 | 3.4 | 6.9 |  |
| COD | CDR | 58 |  | 78 | 4.0 | 64.3 | 92.7 | 51.8 | 30.2 |  | 5.4 | 5.4 | 11.2 | 0.7 | 0.1 | 0.3 |  |
| CIV | Cote d’Ivoire | 54.6 | 10.2 | 62.4 | 17.1 | 80.8 | 92.6 | 48.9 | 29.2 | 5.6 | 5.2 | 5.4 | 10.8 | 3.5 | 0.6 | 1.3 |  |
| DJI | Djibouti | 75.6 |  | 97.5 | 22.0 | 97.5 | 98.5 | 55.3 | 48.6 |  | 8.7 | 8.7 | 18.0 | 15.2 | 2.7 | 5.6 |  |
| GNQ | Equatorial Guinea | 49 |  | 50.4 | 6.7 | 88.3 | 88.6 | 46.7 | 24.0 |  | 4.3 | 4.3 | 8.9 | 1.2 | 0.2 | 0.4 |  |
| ERI | Eritrea | 44 |  | 85.8 | 62.9 | 63.8 | 93.1 | 53.2 | 25.2 |  | 4.5 | 4.5 | 9.3 | 9.0 | 1.6 | 3.3 |  |
| ETH | Ethiopia | 25.8 |  | 94 | 58.9 | 70.7 | 97.9 | 54.7 | 16.4 |  | 2.9 | 2.9 | 6.1 | 7.0 | 1.3 | 2.6 |  |
| GAB | Gabon | 67.4 | 12.5 | 74.8 | 35.3 | 93.1 | 82.5 | 51.2 | 40.8 | 7.7 | 7.3 | 7.6 | 15.1 | 12.8 | 2.3 | 4.7 |  |
| GMB | Gambia | 60.8 |  | 78.1 | 64.0 | 79.7 | 93.9 | 51.8 | 40.8 |  | 7.3 | 7.3 | 15.1 | 18.2 | 3.3 | 6.8 |  |
| GHA | Ghana | 67.5 |  | 75.2 | 35.0 | 90.7 | 94.1 | 51.2 | 43.0 |  | 7.7 | 7.7 | 15.9 | 13.8 | 2.5 | 5.1 |  |
| GIN | Guinea | 52.8 |  | 66.3 | 21.3 | 72.8 | 93.2 | 49.6 | 28.4 |  | 5.1 | 5.1 | 10.5 | 3.7 | 0.7 | 1.4 |  |
| GNB | Guinea-Bissau | 52 |  | 78.6 | 31.6 | 71.8 | 92.9 | 51.9 | 29.5 |  | 5.3 | 5.3 | 10.9 | 6.2 | 1.1 | 2.3 |  |
| KEN | Kenya | 61.8 |  | 78.6 | 43.9 | 77.2 | 93.8 | 51.9 | 37.8 |  | 6.8 | 6.8 | 14.0 | 12.0 | 2.1 | 4.4 |  |
| LBR | Liberia | 76.3 | 18.7 | 72.6 | 69.7 | 70.3 | 94.3 | 50.7 | 47.4 | 12.6 | 8.5 | 11.6 | 17.5 | 18.1 | 3.2 | 6.7 |  |
| MDG | Madagascar | 41.4 | 2.0 | 79.2 | 13.0 | 71.3 | 95.4 | 52.0 | 22.4 | 1.1 | 4.0 | 1.1 | 8.3 | 2.1 | 0.4 | 0.8 |  |
| MWI | Malawi | 57.7 | 16.9 | 84.8 | 90.9 | 73.0 | 90.5 | 53.0 | 37.7 | 11.6 | 6.8 | 11.1 | 14.0 | 21.6 | 3.9 | 8.0 |  |
| MLI | Mali | 49.9 |  | 89.1 | 22.0 | 78.5 | 95.2 | 53.8 | 29.2 |  | 5.2 | 5.2 | 10.8 | 5.8 | 1.0 | 2.1 |  |
| MRT | Mauritania | 45 |  | 85.8 | 6.7 | 74.7 | 96.5 | 53.2 | 24.5 |  | 4.4 | 4.4 | 9.1 | 1.4 | 0.2 | 0.5 |  |
| MOZ | Mozambique | 59.2 | 21.1 | 99.5 | 63.2 | 72.1 | 98.3 | 55.7 | 38.7 | 13.7 | 6.9 | 13.8 | 14.3 | 19.0 | 3.4 | 7.1 |  |
| NAM | Namibia | 58.6 |  | 95.3 | 31.0 | 97.1 | 98.1 | 54.9 | 39.6 |  | 7.1 | 7.1 | 14.7 | 16.0 | 2.9 | 5.9 |  |
| NER | Niger | 61.7 |  | 47.8 | 65.3 | 64.9 | 87.1 | 46.2 | 33.1 |  | 5.9 | 5.9 | 12.3 | 7.1 | 1.3 | 2.6 |  |
| NGA | Nigeria | 83.8 | 3.6 | 41.2 | 11.9 | 83.9 | 89.0 | 45.0 | 40.9 | 2.1 | 7.3 | 1.8 | 15.1 | 2.6 | 0.5 | 1.0 |  |
| RWA | Rwanda | 48.4 | 16.5 | 88.2 | 95.7 | 89.5 | 96.5 | 53.6 | 41.5 | 14.5 | 7.4 | 14.2 | 15.4 | 31.8 | 6.0 | 12.2 |  |
| STP | Sao Tome and Principe | 71.1 |  | 97.5 | 99.2 | 97.5 | 98.5 | 55.3 | 68.8 |  | 12.3 | 12.3 | 25.5 | 65.0 | 16.7 | 29.8 |  |
| SEN | Senegal | 50.4 | 10.6 | 85.8 | 42.7 | 89.1 | 96.5 | 53.2 | 34.0 | 7.3 | 6.1 | 7.2 | 12.6 | 14.2 | 2.5 | 5.3 |  |
| SLE | Sierra Leone | 62 |  | 83.6 | 31.0 | 89.3 | 96.1 | 52.8 | 39.3 |  | 7.0 | 7.0 | 14.6 | 12.4 | 2.2 | 4.6 |  |
| SOM | Somalia | 13 |  | 94 | 59.0 | 64.6 | 97.9 | 54.7 | 7.8 |  | 1.4 | 1.4 | 2.9 | 3.0 | 0.5 | 1.1 |  |
| SDS | South Sudan | 16 |  | 94 | 33.0 | 61.0 | 95.9 | 54.7 | 9.2 |  | 1.7 | 1.7 | 3.4 | 1.1 | 0.2 | 0.4 |  |
| SDN | Sudan | 39.1 |  | 72 | 8.0 | 96.8 | 93.3 | 50.6 | 20.7 |  | 3.7 | 3.7 | 7.7 | 3.2 | 0.6 | 1.2 |  |
| TZA | Tanzania | 77.3 | 13.3 | 73.3 | 55.9 | 72.3 | 92.7 | 50.9 | 46.9 | 8.7 | 8.4 | 8.1 | 17.4 | 15.5 | 2.8 | 5.7 |  |
| TGO | Togo | 34 |  | 76.7 | 28.0 | 78.0 | 95.0 | 51.5 | 19.8 |  | 3.5 | 3.5 | 7.3 | 4.2 | 0.8 | 1.6 |  |
| UGA | Uganda | 84 | 17.4 | 95.3 | 68.6 | 93.9 | 96.9 | 54.9 | 67.1 | 14.9 | 12.0 | 13.9 | 24.9 | 47.0 | 9.9 | 19.2 |  |
| ZMB | Zambia | 68.8 |  | 91.4 | 81.0 | 83.4 | 97.5 | 54.2 | 52.7 |  | 9.4 | 9.4 | 19.5 | 34.7 | 6.7 | 13.6 |  |
| ZWE | Zimbabwe | 42.9 | 15.0 | 85.8 | 49.1 | 75.3 | 96.5 | 53.2 | 27.0 | 9.8 | 4.8 | 9.5 | 10.0 | 9.9 | 1.8 | 3.7 |  |
|  | Unweighted mean | 55.7 |  | 79.6 | 43.6 | 80 | 94.0 | 52.0 | 35.6 | 12.4 | 6.4 | 6.9 | 13.2 | 14.5 | 2.9 | 5.8 |  |
|  | Weighted mean^a^ | 60.5 |  | 71.8 | 35.4 | 79.2 | 93.1 | 50.6 | 35.4 |  | 6.3 | 5.7 | 13.1 | 10.5 | 2.0 | 4.0 |  |
