# Supplementary material for: Effective Coverage and Systems Effectiveness for Malaria Case Management in Sub-Saharan African Countries
Source: PLoS One. 2015 May 22;10(5):e0127818. doi: 10.1371/journal.pone.0127818 (PMC4441512; doi:10.1371/journal.pone.0127818)
Supplement: S2 Table — 1 Access of 32% is estimated from the MICS(2010); 2 Access of 39% is estimated from the MICS(2010); 3 Access of 13% is estimated from the MICS(2008); 4 Access of 30% is estimated from the MICS(2010); 5 Access of 53% is estimated from the MICS(2010); 6 Access of 59.5% is estimated from the MIS(2010); 7 Access of 36.9% is estimated from the MIS(2012). 8 Most estimates are derived from the DHS surveys, and represent proportion of treated fever cases that sought treatment from formal medical care providers including hospitals, health centers, health posts, and community health workers. For countries where DHS surveys were not collected estimates were imputed. The imputation relied on identifying a country with non-missing access data with similar levels of transmission, DTP3 coverage, access to care for fever, per capita health care spending, number of nurses and midwives, percent rural, and population density. Countries were first ordered by level of transmission (low (<10) and high), and then consecutively by each of the other factors, removing most dis-similar records. We weighted stronger similarities in DTP3, access and spending, then the remaining 3 factors. 9 Adherence was imputed for countries for which no information on the parameter could be identified by relating adherence data from the literature and the surveys to per capita health care expenditures. Evidence from the literature suggested uniformly high adherence to ALU across settings. 10 Evidence on parasite drug resistance to non-ACT antimalarials is summarised in [46]. Some of these country estimates are based on trials conducted as far back as 2000; given the rate of resistance we instead applied a median value of parasitological failure rate of sensitive parasites for SP from the survey to all countries. 11 Given the limited geographic coverage of the data a median value over the estimates extracted from the literature were applied to all countries. (DOCX) [file pone.0127818.s006.docx]

| **Country** | **Parameter** | | | | | | |
| --- | --- | --- | --- | --- | --- | --- | --- |
|  | **Access to any provider** | **Access to formal care provider^8^** | **Compliance** | **Adherence^9^** | **Clinical and parasitological failure rate** | **Resistance^10^** | **Incidence of counterfeit and sub-optimal formulations^11^** |
|  | A | Pf | Dpd | Hpd | Cd | Rd | Qpd |
| Angola | DHS(2011) | DHS(2011) | DHS(2011) | DHS(2011) | [1] | [2] | [3] |
| Benin | DHS(2006) | DHS(2006) | [4] |  | [1] | [2] |  |
| Botswana | BFHS(2007) | Imputed from Sao Tome and Principe | [1] |  | [1] | [2] |  |
| Burkina Faso | DHS(2010) | DHS(2010) | DHS(2010) |  | [1] | [2] | [5] |
| Burundi | DHS(2011) | DHS(2011) | DHS(2011) |  | [1] | [2] |  |
| Cameroon | DHS(2011) | DHS(2011) | DHS(2011) | DHS(2011) | [1] | [2] | [6] |
| CAR | DHS(1995) ^1^ | DHS(1995) | MICS(2010) |  | [1] | [2] |  |
| Chad | DHS(2004) | DHS(2004) | MICS(2010) |  | [1] | [2] |  |
| Comoros | MICS(2000) | MICS(2000) | [1] |  | [1] | [2] |  |
| Congo | DHS(2012) | DHS(2012) | DHS(2012) | DHS(2012) | [1] | [2] | [3] |
| CDR | DHS(2007)^2^ | DHS(2007) | MICS(2010) |  | [1] | [2] |  |
| Cote d’Ivoire | DHS(2012) | DHS(2012) | DHS(2012) |  | [1] | [2] |  |
| Djibouti | MIS(2008-2009) | Imputed from Sao Tome and Principe | MICS(2010) |  | [1] | [2] |  |
| Equatorial Guinea | MICS(2000) | Imputed from Cameroon | [1] |  | [1] | [2] |  |
| Eritrea | DHS(2002)^3^ | Imputed from Zimbabwe | [1] |  | [1] | [2] |  |
| Ethiopia | DHS(2011) | DHS(2011) | DHS(2011) | [7] | [1] | [2] | [3,6] |
| Gabon | DHS(2012) | DHS(2012) | DHS(2012) |  | [1] | [2] |  |
| Gambia | MIS(2008)^4^ | MIS(2008) | MICS(2010) |  | [1] | [2] |  |
| Ghana | DHS(2008)^5^ | DHS(2008) | MICS(2010) | DHS(2008) [8] | [1] | [2] | [3,6,9,10] |
| Guinea | DHS(2005) | DHS(2005) | [1] |  | [1] | [2] | [11] |
| Guinea-Bissau | MICS(2010) | Imputed from Kenya | [1] |  | [1] | [2] |  |
| Kenya | DHS(2009)^6^ | DHS(2009) | MIS(2010) | DHS(2009) [12,13] | [1] | [2] | [3,6] |
| Liberia | DHS(2011) | DHS(2011) | DHS(2011) |  | [1] | [2] |  |
| Madagascar | DHS(2011) | DHS(2011) | DHS(2011) |  | [1] | [2] | [14] |
| Malawi | DHS(2012) | DHS(2012) | DHS(2012) | [15] | [1] | [2] |  |
| Mali | MIS(2012) | MIS(2012) | MIS(2012) |  | [1] | [2] |  |
| Mauritania | MICS(2007) | Imputed from Senegal | [1] |  | [1] | [2] |  |
| Mozambique | DHS(2011) | DHS(2011) | DHS(2011) |  | [1] | [2] |  |
| Namibia | DHS(2007)^7^ | DHS(2007) | MIS(2010) |  | [1] | [2] |  |
| Niger | DHS(2006) | DHS(2006) | [1] |  | [1] | [2] |  |
| Nigeria | DHS(2010) | DHS(2010) | [16] | DHS(2010) [8,17] | [1] | [2] | [3,6,9,10,18] |
| Rwanda | DHS(2011) | DHS(2011) | DHS(2011) |  | [1] | [2] | [3] |
| Sao Tome and Principe | DHS(2009) | DHS(2009) | [1] |  | [1] | [2] |  |
| Senegal | DHS(2011) | DHS(2011) | DHS(2011) |  | [1] | [2] | [14] |
| Sierra Leone | MICS(2010) | MICS(2010) | MICS(2010) | DHS(2008) | [1] | [2] |  |
| Somalia | MICS(2006) | Imputed from Ethiopia | [1] |  | [1] | [2] |  |
| South Sudan | [19] | Imputed from Ethiopia | MICS(2010) |  | [1] | [2] |  |
| North Sudan | MIS(2009) | MIS(2009) | MIS(2009) |  | [1] | [2] |  |
| Tanzania | DHS(2012) | DHS(2012) | DHS(2012) | [20–22] | [1] | [2] | [3,6] |
| Togo | MICS(2010) | MICS(2010) | MICS(2010) |  | [1] | [2] | [9] |
| Uganda | DHS(2011) | DHS(2011) | [23] | [8,24–26] | [1] | [2] | [27] [3,14] |
| Zambia | DHS(2007)^7^ | DHS(2007) | [28] | DHS(2007) | [1] | [2] | [3] |
| Zimbabwe | DHS(2011) | DHS(2011) | DHS(2011) |  | [1] | [2] |  |

Reference List

1. World Health Organization (2012) World Malaria Report 2012.

2. World Health Organization (2010) Global report on antimalarial drug efficacy and drug resistance: 2000-2010.

3. Bate R, Zhe Jin G, Mathur A (2011) Does price reveal poor-quality drugs? Evidence from 17 countries. Journal of Health Economics 30: 1150.

4. ACTwatch Group & ABMS/Benin (2013) Household Survey, Benin, 2011 Survey Report.

5. Tipke M, Diallo S, Coulibaly B, Strozinger D, Hoppe-Tichy T, Sie A et al. (2008) Substandard anti-malarial drugs in Burkina Faso. Malaria Journal 7.

6. World Health Organization (2011) Survey of the Quality of Selected Antimalarial Medicines Circulating in Six Countries of Sub-Saharan Africa.

7. Lemma H, Lofrigen C, San Sebastian M (2011) Adherence to a six-dose regimen of artemetherlumefantrine among uncomplicated Plasmodium falciparum patients in the Tigray Region, Ethiopia. Malaria Journal 10: 349.

8. Ajayi FO, Browne EN, Bateganya F, Yar D, Happi C, Falade CO et al. (2008) Effectiveness of artemisinin-based combination therapy used in the context of home management of malaria: A report from three study sites in sub-Saharan Africa. Malaria Journal 7: 190.

9. Bate R, Hess K, Tren R, Mooney L, Cudjoe F, Ayodele T et al. (2012) Subsidizing artemisinin-based combination therapies: a preliminary investigation of the Affordable Medicines Facility- malaria. Research Reports in Tropical Medicine 3: 63-68.

10. Bate R, Hess K (2010) Anti-malarial drug quality in Lagos and Accra-a comparison of various quality assessments. Malaria Journal 9: 157.

11. Klein EY, Lewis IA, Jung C, Llinas M, Levin SA (2012) Relationship between treatment-seeking behaviour and artemisinin drug quality in Ghana. Malaria Journal 11: 110.

12. Ajayi I, Browne E, Garshong B, Bateganya F, Yusuf B, Agyei-Baffour P et al. (2008) Feasibility and acceptability of artemisinin-based combination therapy for the home management of malaria in four African sites. Malaria Journal 7: 6. 10.1186/1475-2875-7-6.

13. Ogolla JO, Ayaya SO, Otieno SA (2013) Levels of adherence to coartem© in the routine treatment of uncomplicated malaria in children aged below five years, in Kenya. Iranian Journal of Public Health 42: 129-133.

14. US Pharmacopeia (2009) Survey of the quality of selected antimalarial medicines circulating in Madagascar, Senegal, and Uganda.

15. Mace KE, Mwandama D, Jafali J, Lika M, Filler SJ, Sande J et al. (2011) Adherence to treatment with artemether-lumefantrine for uncomplicated malaria in rural Malawi. Clinical Infectious Diseases 53: 772-779.

16. ACTwatch Group & SFN/Nigeria (2013) Household Survey, Nigeria, 2012 Survey Report.

17. Meremikwu M, Odey F, Oringanje C, Oyo-Ita A, Udoh E, Eyong K et al. (2013) Effectiveness of a 6-dose regimen of Artemether-Lumefantrine for unsupervised treatment of uncomplicated childhood malaria in Calabar, Nigeria. Nigerian Journal of Paediatrics 40: 145-149.

18. Onwujekwe O, Kaur H, Dike N, Shu E, Uzochukwu BSC, Hanson K et al. (2009) Quality of anti-malarial drugs provided by public and private healthcare providers in south-east Nigeria. Malaria Journal 8.

19. Wakabi W (2011) South Sudan faces grim health and humanitarian situation. The Lancet 377: 2167-2168.

20. Beer N, Ali AS, Rotllant G, Abass AK, Omari RS, Al-mafazy AH et al. (2009) Adherence to artesunate-amodiaquine combination therapy for uncomplicated malaria in children in Zanzibar, Tanzania. Tropical Medicine and International Health 14: 766-774.

21. Simba DO, Kakoko D, Tomson G, Premji Z, Petzold M, Mahindi M et al. (2012) Adherence to artemether/lumefantrine treatment in children under real-life situations in rural Tanzania. Transactions of the Royal Society of Tropical Medicine and Hygiene 106: 3-9.

22. Kabanywanyi AM, Lengeler C, Kasim P, King'eng'ena S, Schlienger R, Mulure N et al. (2010) Adherence to and acceptability of artemether-lumefantrine as first-line anti-malarial treatment: evidence from a rural community in Tanzania. Malaria Journal 9: 48.

23. ACTwatch Group & PACE/Uganda (2013) Household Survey, Uganda, 2012 Survey Report.

24. Fogg C, Bajunirwe F, Piola P, Biraro S, Checchi F, Kiguli J et al. (2004) Adherence to a six-dose regimen of artemether-lumefantrine for treatment of uncomplicated Plasmodium Falciparum malaria in Uganda. American Journal of Tropical Medicine and Hygine 71: 525-530.

25. Cohen J, Yavuz E, Morris A, Arkedis J, Sabot O (2012) Do patients adhere to over-the-counter artemisinin combination therapy for malaria? evidence from an intervention study in Uganda. Malaria Journal 11: 83. 10.1186/1475-2875-11-83.

26. Kalyango JN, Rutebemberwa E, Karamagi C, Mworozi E, Ssali S, Alfven T et al. (2013) High Adherence to Antimalarials and Antibiotics under Integrated Community Case Management of Illness in Children Less than Five Years in Eastern Uganda. PLoS One 8: e60481.

27. Bjorkman-Nyqvist M, Svensson J, Yanagizawa-Drott D (2013) The Market for (Fake) Antimalarial Medicine: Evidence from Uganda.

28. ACTwatch Group & SFH/Zambia (2013) Household Survey, Zambia, 2011 Survey Report.
