# Supplementary material for: Effective Coverage and Systems Effectiveness for Malaria Case Management in Sub-Saharan African Countries
Source: PLoS One. 2015 May 22;10(5):e0127818. doi: 10.1371/journal.pone.0127818 (PMC4441512; doi:10.1371/journal.pone.0127818)
Supplement: S3 Table — (DOCX) [file pone.0127818.s007.docx]

| Country | Source | Survey year | Drug | Unit | Rural Urban | Point of sale | Sampling | Sample size | Population | Defined as adherent | Proportion adhered with drug regimen | | |
| --- | --- | --- | --- | --- | --- | --- | --- | --- | --- | --- | --- | --- | --- |
|  |  |  |  |  |  |  |  |  |  |  | All | Formal | Informal |
| Angola | DHS | 2011 | ALU | National |  |  | National | 566 | <5 | Taken drug for at least 3 days | 0.94 |  |  |
| Cameroon | DHS | 2011 | ALU | National |  |  | National | 135 | <5 | Taken drug for at least 3 days | 0.89 | 0.94 | 0.93 |
| Congo | DHS | 2005 | ALU | National |  |  | National | 61 | <5 | Taken drug for at least 3 days | 0.98 |  |  |
| Ethiopia | [1] | 2008 | ALU | District | Rural | Health post | Convenience | 209 |  | Blister count ; Taken drug for at least 3 days |  | 0.39 |  |
| Ghana | DHS | 2008 | ALU | National |  |  | National | 6 | <5 | Taken ACT for 3 days | 1.00 |  |  |
| Ghana | [2] | 2004 | ASAQ | District | Rural | Community medicine distributors | Convenience | 211 |  | Correct dose; Taken ACT for 3 days |  |  | 0.97 |
| Kenya | DHS | 2009 | ALU | National |  |  | National | 94 | <5 | Taken ACT for at least 3 days | 0.89 | 0.94 | 0.27 |
| Kenya | [3] | 2010 | ALU |  |  | Dispensary, health center |  | 646 |  | Blister count ; Taken drug for 3 days; 4 day follow-up |  |  | 0.64 |
| Kenya | [3] | 2001 | ALU |  |  | Dispensary |  | 272 |  | Blister count ; Taken drug for 3 days; 4 day follow-up |  |  | 0.65 |
| Kenya | [4] | 2009 | ALU | District |  | Hospital | Convenience | 62 | <5 | Blister count ; Taken drug for 3 days |  | 0.76 |  |
| Malawi | [5] | 2007 | ALU | District | Rural | Health center | Convenience | 386 |  | Blister count ; Dose recall; 72 hours |  | 0.75 |  |
| Nigeria | DHS | 2010 | ALU | National |  |  | National | 103 | <5 | Taken ACT for at least 3 days | 0.82 | 0.89 | 0.71 |
| Nigeria | [2] | 2004 | ALU | District | Rural | Community medicine distributors | Convenience | 132 |  | Correct dose; Taken ACT for 3 days |  |  | 0.93 |
| Nigeria | [6] | 2007 | ALU | District | Urban | Hospital | Convenience | 215 |  | Blister count |  | 0.98 |  |
| Sierra Leone | DHS | 2008 | ALU | National |  |  | National | 37 | <5 | Taken ACT for at least 3 days | 0.91 |  |  |
| Tanzania | [7] | 2009 | ASAQ | District(8) |  | Health center | Stratified cluster | 210 | <5 | Blister count ; Taken drug for 3 days |  | 0.77 |  |
| Tanzania | [8] | 2008 | ALU | District | Rural | Health center | Stratified cluster | 444 | <5 | Drug administered by care taker for 2 days; 7 day follow-up |  | 0.88 |  |
| Tanzania | [9] | 2008 | ALU | District | Rural | Health center |  | 522 |  | Taken 3 doses |  | 0.98 |  |
| Uganda | [10] | 2002 | ALU | District | Semi-urban | Hospital, dispensary | Convenience | 235 |  | Blister count ; Self-reported adherence |  | 0.90 |  |
| Uganda | [2] | 2004 | ALU | District | Rural | Community medicine distributors | Convenience | 44 |  | Correct dose; Taken ACT for 3 days |  |  | 0.81 |
| Uganda | [11] | 2009 | ALU | District | Rural | Dispensary | Convenience | 395 |  | Blister count ; Taken drug for 3 days; 3 day follow-up |  |  | 0.66 |
| Uganda | [12] | 2011 | ALU | District | Rural | Community health worker | Cluster randomized | 667 | <5 | Blister count ; Taken drug for 3 days; 4 day follow-up |  | 0.96 |  |
| Zambia | DHS | 2007 | ALU | National |  |  | National | 115 | <5 | Taken ACT for at least 3 days | 0.93 |  |  |

Reference List

1. Lemma H, Lofrigen C, San Sebastian M (2011) Adherence to a six-dose regimen of artemetherlumefantrine among uncomplicated Plasmodium falciparum patients in the Tigray Region, Ethiopia. Malaria Journal 10: 349.

2. Ajayi I, Browne E, Garshong B, Bateganya F, Yusuf B, Agyei-Baffour P et al. (2008) Feasibility and acceptability of artemisinin-based combination therapy for the home management of malaria in four African sites. Malaria Journal 7: 6. 10.1186/1475-2875-7-6.

3. Lawford H, Zurovac D, O`Reilly L, Hoibak S, Cowley A, Munga S et al. (2011) Adherence to prescribed artemisinin-based combination therapy in Garissa and Bunyala districts, Kenya. Malaria Journal 10.

4. Ogolla JO, Ayaya SO, Otieno SA (2013) Levels of adherence to coartem© in the routine treatment of uncomplicated malaria in children aged below five years, in Kenya. Iranian Journal of Public Health 42: 129-133.

5. Mace KE, Mwandama D, Jafali J, Lika M, Filler SJ, Sande J et al. (2011) Adherence to treatment with artemether-lumefantrine for uncomplicated malaria in rural Malawi. Clinical Infectious Diseases 53: 772-779.

6. Meremikwu M, Odey F, Oringanje C, Oyo-Ita A, Udoh E, Eyong K et al. (2013) Effectiveness of a 6-dose regimen of Artemether-Lumefantrine for unsupervised treatment of uncomplicated childhood malaria in Calabar, Nigeria. Nigerian Journal of Paediatrics 40: 145-149.

7. Beer N, Ali AS, Rotllant G, Abass AK, Omari RS, Al-mafazy AH et al. (2009) Adherence to artesunate-amodiaquine combination therapy for uncomplicated malaria in children in Zanzibar, Tanzania. Tropical Medicine and International Health 14: 766-774.

8. Simba DO, Kakoko D, Tomson G, Premji Z, Petzold M, Mahindi M et al. (2012) Adherence to artemether/lumefantrine treatment in children under real-life situations in rural Tanzania. Transactions of the Royal Society of Tropical Medicine and Hygiene 106: 3-9.

9. Kabanywanyi AM, Lengeler C, Kasim P, King'eng'ena S, Schlienger R, Mulure N et al. (2010) Adherence to and acceptability of artemether-lumefantrine as first-line anti-malarial treatment: evidence from a rural community in Tanzania. Malaria Journal 9: 48.

10. Fogg C, Bajunirwe F, Piola P, Biraro S, Checchi F, Kiguli J et al. (2004) Adherence to a six-dose regimen of artemether-lumefantrine for treatment of uncomplicated Plasmodium Falciparum malaria in Uganda. American Journal of Tropical Medicine and Hygine 71: 525-530.

11. Cohen J, Yavuz E, Morris A, Arkedis J, Sabot O (2012) Do patients adhere to over-the-counter artemisinin combination therapy for malaria? evidence from an intervention study in Uganda. Malaria Journal 11: 83. 10.1186/1475-2875-11-83.

12. Kalyango JN, Rutebemberwa E, Karamagi C, Mworozi E, Ssali S, Alfven T et al. (2013) High Adherence to Antimalarials and Antibiotics under Integrated Community Case Management of Illness in Children Less than Five Years in Eastern Uganda. PLoS One 8: e60481.
