# Supplementary material for: Effective Coverage and Systems Effectiveness for Malaria Case Management in Sub-Saharan African Countries
Source: PLoS One. 2015 May 22;10(5):e0127818. doi: 10.1371/journal.pone.0127818 (PMC4441512; doi:10.1371/journal.pone.0127818)
Supplement: S4 Table — (DOCX) [file pone.0127818.s008.docx]

| Source | Survey Year | Country | Scope | Rural Urban | Point of Sale | Drug Tested | | Method of Testing | Sampling | Samples Tested | Percent Counterfeit |
| --- | --- | --- | --- | --- | --- | --- | --- | --- | --- | --- | --- |
| [1] | 2010 | Uganda | District |  | Drug store | ACT | | Raman | Random |  | 14.1% |
| [1] | 2010 | Uganda | District |  | Drug store | ACT | | Raman | Random |  | 11.1% |
| [1] | 2010 | Uganda | District |  | Drug store | ACT | | Raman | Random |  | 30%% |
| [1] | 2010 | Uganda | District |  | Drug store | ACT | | Raman | Random |  | 25.6% |
| [2] | 2009 2011 | Angola China Congo Egypt Ethiopia Ghana India Kenya Nigeria Rwanda Tanzania Thailand Uganda Zambia |  | Urban | Private pharmacy | ACT | | Visual, Minilab, Raman | Convenience | 79 | 14% |
| [3] | 2008 | Nigeria | Town | Urban | All level providers | | DHA | HPLC, dissolution | Random | 4 | 25% |
| [4] | 2011 | Nigeria | Town | Urban | Private pharmacy | | ALU | TLC, HPLC |  | 44 | 2.3% |
| [4] | 2011 | Ghana | Town | Urban | Private pharmacy | | ALU | TLC, HPLC |  | 13 | 7.7% |
| [4] | 2011 | Ghana | Town | Urban | Private pharmacy | | ALU | TLC, HPLC |  | 27 | 0% |
| [4] | 2011 | Togo | Town | Urban | Private pharmacy | | ALU | TLC, HPLC |  | 9 | 0% |
| [5] | 2006 | Burkina Faso | District |  | Private pharmacies | | ALU | TCL, QC, visual, dissolution | Convenience | 4 | 0% |
| [6] | 2008 | Madagascar | District(3) |  | Wholesale, retail outlets | | ACT | GPHF-Minilab, QC, visual, dissolution | Convenience | 32 | 16% |
| [6] | 2008 | Uganda | Region |  | Wholesale, retail outlets | | ACT | GPHF-Minilab, QC, visual, dissolution | Convenience | 39 | 36% |
| [6] | 2008 | Senegal | District(8) |  | Wholesale, retail outlets | | ACT | GPHF-Minilab, QC, visual, dissolution | Convenience | 35 | 40% |
| [7] | 2008 | Cameroon |  |  | Wholesale, retail outlets | | ACT | TLC, QC, visual, dissolution | Convenience | 12 | 32% |
| [7] | 2008 | Ethiopia |  |  | Wholesale, retail outlets | | ACT | TLC, QC, visual, dissolution | Convenience | 14 | 0% |
| [7] | 2008 | Ghana |  |  | Wholesale, retail outlets | | ACT | TLC, QC, visual, dissolution | Convenience | 10 | 27% |
| [7] | 2008 | Kenya |  |  | Wholesale, retail outlets | | ACT | TLC, QC, visual, dissolution | Convenience | 23 | 9% |
| [7] | 2008 | Nigeria |  |  | Wholesale, retail outlets | | ACT | TLC, QC, visual, dissolution | Convenience | 20 | 62% |
| [7] | 2008 | Tanzania |  |  | Wholesale, retail outlets | | ACT | TLC, QC, visual, dissolution | Convenience | 15 | 1% |
| [8] | 2010 | Ghana | Town | Peri-urban | Pharmacy, market, shops, sellers | | ALU | NMR, visual, dissolution | Convenience | 15 | 7% |
| [9] | 2010 | Nigeria | Town | Urban | Pharmacy | | ACT | Raman | Convenience | 15 | 7% |
| [9] | 2010 | Ghana | Town | Urban | Pharmacy | | ACT | Raman | Convenience | 8 | 23% |
|  |  |  |  |  |  | |  |  |  |  |  |

Reference List

1. Bjorkman-Nyqvist M, Svensson J, Yanagizawa-Drott D (2013) The Market for (Fake) Antimalarial Medicine: Evidence from Uganda.

2. Bate R, Zhe Jin G, Mathur A (2011) Does price reveal poor-quality drugs? Evidence from 17 countries. Journal of Health Economics 30: 1150.

3. Onwujekwe O, Kaur H, Dike N, Shu E, Uzochukwu BSC, Hanson K et al. (2009) Quality of anti-malarial drugs provided by public and private healthcare providers in south-east Nigeria. Malaria Journal 8.

4. Bate R, Hess K, Tren R, Mooney L, Cudjoe F, Ayodele T et al. (2012) Subsidizing artemisinin-based combination therapies: a preliminary investigation of the Affordable Medicines Facility- malaria. Research Reports in Tropical Medicine 3: 63-68.

5. Tipke M, Diallo S, Coulibaly B, Strozinger D, Hoppe-Tichy T, Sie A et al. (2008) Substandard anti-malarial drugs in Burkina Faso. Malaria Journal 7.

6. US Pharmacopeia (2009) Survey of the quality of selected antimalarial medicines circulating in Madagascar, Senegal, and Uganda.

7. World Health Organization (2011) Survey of the Quality of Selected Antimalarial Medicines Circulating in Six Countries of Sub-Saharan Africa.

8. Klein EY, Lewis IA, Jung C, Llinas M, Levin SA (2012) Relationship between treatment-seeking behaviour and artemisinin drug quality in Ghana. Malaria Journal 11: 110.

9. Bate R, Hess K (2010) Anti-malarial drug quality in Lagos and Accra-a comparison of various quality assessments. Malaria Journal 9: 157.
